# RAPTURE (RAD capture) panel facilitates analyses characterizing sea lamprey reproductive ecology and movement dynamics

**DOI:** 10.1002/ece3.6001

**Published:** 2020-01-15

**Authors:** Nicholas M. Sard, Seth R. Smith, Jared J. Homola, Jeannette Kanefsky, Gale Bravener, Jean V. Adams, Christopher M. Holbrook, Peter J. Hrodey, Kevin Tallon, Kim T. Scribner

**Affiliations:** ^1^ Department of Fisheries and Wildlife Michigan State University East Lansing Michigan; ^2^ Biology Department SUNY Oswego Oswego New York; ^3^ Fisheries and Oceans Canada Sault Ste. Marie ON Canada; ^4^ Great Lakes Science Center U.S. Geological Survey Ann Arbor Michigan; ^5^ Great Lakes Science Center Hammond Bay Biological Station U.S. Geological Survey Millersburg Michigan; ^6^ U.S. Fish and Wildlife Service Marquette Michigan; ^7^ Department of Integrative Biology State University East Lansing Michigan

**Keywords:** Great Lakes, pedigree reconstruction, population structure, RAD capture, Sea lamprey

## Abstract

Genomic tools are lacking for invasive and native populations of sea lamprey (*Petromyzon marinus*). Our objective was to discover single nucleotide polymorphism (SNP) loci to conduct pedigree analyses to quantify reproductive contributions of adult sea lampreys and dispersion of sibling larval sea lampreys of different ages in Great Lakes tributaries. Additional applications of data were explored using additional geographically expansive samples. We used restriction site‐associated DNA sequencing (RAD‐Seq) to discover genetic variation in Duffins Creek (DC), Ontario, Canada, and the St. Clair River (SCR), Michigan, USA. We subsequently developed RAD capture baits to genotype 3,446 RAD loci that contained 11,970 SNPs. Based on RAD capture assays, estimates of variance in SNP allele frequency among five Great Lakes tributary populations (mean *F*_ST_ 0.008; range 0.00–0.018) were concordant with previous microsatellite‐based studies; however, outlier loci were identified that contributed substantially to spatial population genetic structure. At finer scales within streams, simulations indicated that accuracy in genetic pedigree reconstruction was high when 200 or 500 independent loci were used, even in situations of high spawner abundance (e.g., 1,000 adults). Based on empirical collections of larval sea lamprey genotypes, we found that age‐1 and age‐2 families of full and half‐siblings were widely but nonrandomly distributed within stream reaches sampled. Using the genomic scale set of SNP loci developed in this study, biologists can rapidly genotype sea lamprey in non‐native and native ranges to investigate questions pertaining to population structuring and reproductive ecology at previously unattainable scales.

## Graphical abstract

We developed a single nucleotide polymorphism (SNP) panel for pedigree analyses for larval sea lamprey to characterize levels of diversity within and among populations (mean interpopulation *F*_ST_ range 0.001–0.020). Genome‐wide annotation based on the sea lamprey genome facilitated the identification of putative functions of outlier loci (range of interpopulation *F*_ST_ 0.205–0.374). Simulation and empirical analyses demonstrated accuracy in pedigree assignment, and facilitated estimation of effective breeding population size, aspects of the species reproductive ecology, and extent of larval dispersal.

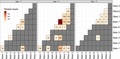

## 1 INTRODUCTION

Over the past decade, several technological and methodological advances have dramatically increased the number of loci available to study a range of ecological and evolutionary questions. The additional information gained has substantially increased the precision and accuracy of population genetic inference (e.g., estimation of relatedness, inbreeding, spatial structure, Barth et al., 2017; Kardos, Luikart, & Allendorf, 2015; Kardos, Taylor, Ellegren, Luikart, & Allendorf, 2016) and enabled locus‐specific analyses (e.g., outlier tests, association analyses; Allendorf, Hohenlohe, & Luikart, 2010; Luikart, England, Tallmon, Jordon, & Taberlet, 2003). Research and monitoring of nonmodel organisms that employ expansive suites of loci are increasingly used in combination with demographic data over spatial and temporal scales previously not possible (e.g., Waples et al., 2018).

Genomic data are increasingly used in fisheries research and monitoring (e.g., Hand et al., 2015; Hess et al., 2014; Hohenlohe et al., 2013). For example, researchers are using genomic data in fish populations to identify genes and genomic regions associated with phenotypes and fitness in different environments (Cosart et al., 2011; Hand et al., 2016; Hohenlohe et al., 2013; Prince et al., 2017). Genotype‐by‐sequencing methods have also been applied in large‐scale genetic pedigree reconstruction (e.g., parentage‐based tagging, Anderson & Garza, 2006; Campbell, Harmon, & Narum, 2015: Beacham et al., 2017; close‐kin capture–mark–release analyses, Baetscher et al., 2019) to characterize aspects of species reproductive ecology, demography, and dispersal during early life stages.

Sea lamprey (*Petromyzon marinus*) are an anadromous fish native to the Atlantic Ocean and can be found in North American and European waters (Beamish, 1980). Currently, several sea lamprey populations in Europe are considered threatened (Mateus, Rodríguez‐Muñoz, Quintella, Alves, & Almeida, 2012). Following the opening of the Welland Canal in 1829 (Morman, Cuddy, & Rugen, 1980), sea lamprey and many other non‐native species invaded the North American Great Lakes, and by 1946, sea lamprey had invaded all five Great Lakes (Christie, 1974; Lawrie, 1970). The introduction of sea lamprey into the Great Lakes exposed large piscivorous native fish species to a parasitic predator and led to catastrophic declines in abundance of ecologically and economically important species, including the top predators (Smith & Tibbles, 1980). An extensive control program, conducted since the late 1950s, has generated considerable information about the species' life cycle and ecology (Applegate, 1950; Hansen et al., 2016). Like other anadromous fishes, adult sea lamprey spawn in streams. Filter‐feeding larvae typically live for two to five years before transforming into parasitic juveniles and migrating to the sea or lake in search of a host. In the Great Lakes, parasitic sea lampreys typically spend 12–18 months in the lake before migrating back into streams to spawn. Unlike many other anadromous fishes, sea lampreys do not home to natal streams (Bergstedt & Seelye, 1995; Waldman, Grunwald, Roy, & Wirgin, 2004) but use chemical cues (pheromones) from larval lampreys to identify streams with suitable spawning and rearing habitat (Bjerselius et al., 2000; Sorensen & Vrieze, 2003), resulting in highly panmictic populations within lakes.

In the Great Lakes, sea lamprey control has been largely dependent on annual lampricide treatments that kill larvae in streams and dams that prevent adults from accessing spawning habitat (Applegate, Howell, Moffett, & Smith, 1961; Christie et al., 2003). The status of sea lamprey populations and success of the control program as whole are determined largely from mark–recapture methods to estimate stream‐specific adult sea lamprey abundance from trap catches (Mullett et al., 2003) and prey wounding estimates from native lake trout (*Salvelinus namaycush*) populations (Rutter & Bence, 2003). Though sea lamprey populations are currently below historic (postinvasion) levels, abundance and prey wounding estimates have exceeded target levels in some lakes in recent years (Sullivan, Adair, & Woldt, 2016). Annual assessments in several larger Great Lakes tributaries and connecting waterways have revealed that sizable numbers of outmigrating juveniles have been produced (Sullivan et al., 2016), yet the sources of those lampreys are unknown. Sea lamprey demographic data, specifically adult abundance in untrappable streams and stream‐specific recruitment, are needed to track the status of these populations. Capture–mark–recapture methods employing traps are often used to estimate the number of adults (Mullett et al., 2003), but trapping in large nonwadable rivers is not feasible with conventional methods due to the rivers’ large width, length, depth, and flow. Genetic data and genetic pedigree reconstruction methods may be a valuable alternative approach for estimating adult sea lamprey abundance in Great Lakes tributaries, as has been demonstrated with other fishes in large systems (e.g., Abadia‐Cardoso, Anderson, Pearse, & Garza, 2013; Bravington, Eveson, Grewe, & Davies, 2017; Steele et al., 2013). Studies of native lamprey in support of conservation initiatives involving parentage analysis include Pacific lamprey (*Lampetra tridentata*; Hess et al., 2015) and European River lamprey (*Lampetra fluviatilis*; Rougemont et al., 2015; Hume, Rechnagel, Bean, Adams, & Mable, 2018).

Genomic scale tools are lacking for invasive and native populations of sea lamprey throughout their range. Current genetic assays that are routinely employed for sea lamprey in the Great Lakes (e.g., microsatellite DNA genotyping; Bryan, Libants, Warrillow, Li & Scribner, 2003, Filcek, Gilmore, Jones, & Scribner, 2005 or mtDNA; Waldman et al., 2004) are not adequate to meet management needs because they lack sufficient power to answer high priority questions (e.g., SLCB, 2016). For example, existing data and the number of genetic markers available can be used for sea lamprey on small spatial scales or experimentally (e.g., Dawson, Jones, Scribner, & Gilmore, 2009; Derosier, Jones, & Scribner, 2007) but are insufficient to accurately determine parentage or pedigree relationships in large Great Lakes tributary systems where large numbers (1,000s or 10,000s) of adults could be spawning. However, others have shown that similar inferences are possible using genomic data (e.g., Baetscher, Clemento, Ng, Anderson, & Garza, 2017; Strucken et al., 2016).

A number of molecular techniques now allow targeted sequencing of hundreds or thousands of loci and samples at relatively low cost. A spatially referenced and standardized Great Lakes sea lamprey genomics database that ties larval pedigrees to locations could assist managers in achieving monitoring and sea lamprey suppression goals for invasive Great Lakes populations in North America. Applications for invasive populations include improved larval assessment, enumeration of spawning adult abundance, identification of genes associated with physiological pathways under selection, and use of genetic control methods (McCauley, Docker, Whyard, & Li, 2015). Applications for conservation of anadromous populations in Europe and the Atlantic coastal populations of North America include investigations of and characterization of effective population size (Waples, 2010; Waples, Larson, & Waples, 2016), the study of local adaptation (Savolainen, Lascoux, & Merila, 2013) important for reintroductions, or simply be used in the context of genetic monitoring of reintroductions (Sard et al., 2016). Analyses could be further enabled by the existence of an annotated sea lamprey genome (Smith et al., 2018).

Protocols that combine targeted sequence capture with restriction site‐associated DNA sequencing (Ali et al., 2016; Hoffberg et al., 2016) and highly multiplexed amplicon sequencing protocols (GT‐Seq; Campbell et al., 2015) are beginning to be widely used for conservation genomic applications (Meek & Larson, 2019). Greater understanding of sea lamprey mating ecology and movement dynamics of sibling groups prior to metamorphosis could greatly aid sea lamprey control efforts. Thus, the overarching project objective of this study was to develop a panel of SNPs for sea lamprey and to apply the multilocus SNP panel to conduct genetic pedigree analyses of larval sea lampreys to characterize aspects of the species mating ecology and dispersal behavior. Specific objectives were to (a) estimate larval membership to half‐ and full‐sibling family groups and the number of adult sea lampreys that produced larvae captured in two Great Lakes tributaries representing extreme ranges in size and sampling accessibility, (b) determine sea lamprey spawning locations, and range of downstream larval dispersal in the St. Clair River based on locations of siblings, and (c) characterize levels of spatial genetic structure among Great Lakes tributaries.

## 2 METHODS

### 2.1 Sample field collections

**Figure 1:**
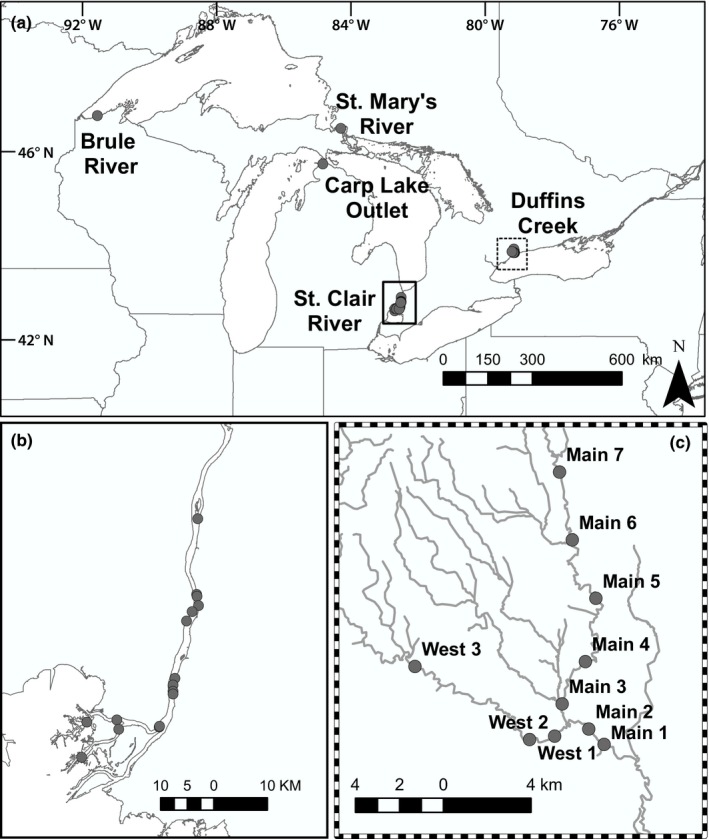
Map showing locations of Great Lakes tributaries where sea lamprey were collected (a), including larvae in the St. Clair River (SCR), Michigan, USA (b), and in Duffins Creek (DC), Ontario, Canada (c). Larval sampling locations in SCR and DC are indicated by dots. Adults were sampled in the Brule River, St. Mary's River, and Carp Lake Outlet. Locations in the St. Clair were not numbered in the map, like Duffins Creek, because no siblings were identified, and thus, similar analyses (e.g., Figure 7) could not be conducted

Larval sea lampreys were collected from the St. Clair River (SCR) and Duffins Creek (DC) (Figure 1) by U.S. Fish and Wildlife Service (USFWS), and Department of Fisheries and Oceans Canada (DFO) staff, respectively, in May and June of 2017 during routine assessment sampling. Samples were collected by applying granular bayluscide to nonwadable waters (SCR; 2.6 hectare area) which allowed larvae to be collected downstream in drift nets, or using ABP‐2 backpack electrofishers in wadable waters (DC).Sampling locations in DC were chosen so that the spatial extent of the larval sea lamprey distribution was well represented (see map in Figure 1). All individuals were retained irrespective of size/age, and therefore, because of the area sampled, samples were expected to include a broad range of ages and family groups. Samples (whole body) were stored in 95% ethanol and delivered to the laboratory at Michigan State University (MSU). In the laboratory, we measured body length for each individual from DC and used a mixture of four Gaussian distributions to group larval body lengths into age 0, age 1, and age 2, age 3+ years (Sethi, Gerken, & Ashline, 2017). We used the DC length–frequency distributions to age larvae from the SCR because of small sample size (Table 1). To facilitate exploration of other analyses focusing on spatial genetic structure, samples were also collected from widely dispersed spawning adult sea lampreys from Great Lakes tributaries by USFWS staff (Brule River in the Lake Superior basin [*n* = 25], Carp River in the Lake Michigan basin [*n* = 15], St. Mary's River in the Lake Huron basin [*n* = 25], Table 1 and Figure 1).

**Table 1:** Sample sizes genotyped in discovery and RAD capture libraries and the downsampled dataset of unrelated individuals by sampling location and age

Location	Age	Libraries		Downsampled dataset
		Discovery	RAPTURE	
Duffins Creek	0	10	10	—
	1	47	47	8
	2	162	162	23
	3+	32	32	14
St. Clair River	0	1	1	—
	1	1	1	—
	2	19	19	15
	3+	16	16	15
Brule River	Adult	—	25	25
Carp River	Adult	—	15	15
St. Mary's River	Adult	—	25	25

### 2.2 Morphological and genetic verification of species identity

Field crews distinguished larval sea lampreys from larvae belonging to native lamprey species (*Ichthyomyzon* spp., *Lampetra appendix*) using morphometric features including trunk myomere counts and pigment location and density. We also used a genetic method to verify species identity. DNA was extracted from 288 larval sea lampreys collected in the SCR (*n* = 37, Table 1) and DC (*n* = 251, Table 1) using Qiagen DNeasy Kits (Qiagen, Valencia, CA) to validate field species identification using sea lamprey specific cytochrome *c* oxidase subunit I (COI) single‐species DNA barcoding PCR primers (Gingera et al., 2016). At the time DNA was extracted from samples, body weight and total length were recorded. PCRs were carried out as described in Gingera et al. (2016) and contained 20 ng of DNA at a concentration of 10 ng/µl and 23 µl of master mix (2.5 µl of Amplitaq Gold PCR Buffer II‐ no Mg+2, 1.5 µl of 25 mM MgCl_2_, 2.5 µl of 2 mM dNTPs, 0.5 µl each of the sea lamprey forward (5′‐GGCAACTGACTTGTACCMCTAATACTTGGT‐3′) and reverse (5′‐ GGCTAAGTGTAAGGAAAAGATTGTTAGGTCGAC‐3′) primers at 10 pmol/µl, 15.37 µl of diH2O and 0.13 µl of Amplitaq Gold (Applied Biosystems) at 5 U/µl) for a total volume of 25 µl. Cycling conditions were as follows: an initial denaturation step of 5 min at 95°C, followed by 35 cycles of 30 s at 95°C, 30 s at 55°C and 30 s at 72°C, and a final extension step of 5 min at 72°C. PCR products (expected size of 225 bp) were run on 1.5% agarose gels along with a 100 bp DNA ladder (Invitrogen) and stained with ethidium bromide for visualization. All PCRs were conducted included negative (no DNA) and positive (known DNA from adult sea lamprey) controls.

### 2.3 Genomic library preparation

#### 2.3.1 Sample preparation and locus discovery using RAD sequencing

DNA samples extracted from larval sea lamprey from DC and SCR were used to construct RAD libraries used for SNP discovery. Double‐stranded DNA concentrations were determined using Quant‐iT PicoGreen assays (Thermo Scientific), and samples were normalized to a concentration of 10 ng/µl before proceeding with RAD library preparation and sequencing.

Restriction site‐associated DNA (RAD) libraries were prepared following the “BestRAD” protocol described in Ali et al. (2016). After normalizing DNA concentrations, DNA was digested using high fidelity *Sbf*I (New England Biolabs). After 60 min of digestion at 37°C and a 20‐min inactivation step at 65°C, BestRAD adapters were ligated to *Sbf*I cut site overhangs using T4 Ligase (New England Biolabs). For each 96‐well plate of samples, equal volumes of adapter ligated DNA were pooled for each sample and adapter dimers were removed using Ampure XP beads (Beckman Coulter; 1.2:1 ratio beads to DNA). The multiplexed libraries were then eluted in 130 µl Tris‐EDTA buffer and sheared using a Covaris M220 Ultrasonicator (Covaris) to an average fragment length of approximately 550 bp. Sheared and barcoded RAD loci were then isolated using the streptavidin bead‐binding assay described in Ali et al. (2016). Illumina adapters were added to isolated fragments using the NEBNext Library Prep Kit for Illumina (New England Biolabs), 1:10 diluted Y‐adapters, and the optional size‐selection step. Libraries were dual‐indexed using NEB Dual Index Set 1 and amplified for 12 cycles before being purified using Ampure XP beads (0.9:1 ratio beads to DNA) and eluted in low EDTA TE. Libraries were quantified using Quant‐iT PicoGreen assays run in triplicate.

For the discovery libraries, four libraries were sequenced on a single paired‐end 150 base pair HiSeq 4000 lane at the MSU Research Technology Support Facility. STACKS v1.44 was used to process the 332 million read pairs using all default parameters (Catchen, Amores, Hohenlohe, Cresko, & Postlethwait, 2011; Catchen, Hohenlohe, Bassham, Amores, & Cresko, 2013), except in cstacks ‐n was set to two. We preformed de novo assembly first, as recommended by Paris, Stevens, and Catchen (2017). When forming the catalog, we used 15 individuals from each population that had the highest number of total reads. We allowed one mismatch between observed and expected barcodes and restriction site sequences during process_radtags. In addition, reads with uncalled bases or low‐quality reads (default STACKS value: average phred score of 10 in a 22 bp sliding window across the read) were removed. PCR duplicates were removed using clone_filter to avoid inflated coverages that could lead to incorrect genotype calling (Andrews, 2014). Analyses were performed using MSU's Institute for Cyber Enabled Research (iCER) High‐Performance Computing Cluster (HPCC). Loci were included in the final variant call format (VCF) file if they were genotyped in 90% of individuals at 10× coverage for all larval samples. Additional filtering of putative paralogs and loci with excessively high observed heterozygosity (>0.60) and *z*‐scores less than or greater than 7 were done using the program HDplot (McKinney, Waples, Seeb, & Seeb, 2017).

#### 2.3.2 RAD capture methods

Using the SNP discovery dataset described above, we designed RNA baits needed to apply the targeted RAD capture (RAPTURE) sequencing approach described by Ali et al. (2016). Consensus sequences for variable RAD loci identified above (output by the STACKs “populations” module) were aligned to the sea lamprey genome (Smith et al., 2018) using BWA‐mem (Li, 2013). RAD loci mapping to multiple locations were excluded from the dataset using SAMtools (Li et al., 2009). Mapping coordinates for the remaining loci were lengthened by 100 base pairs using bedtools slopBed, and extended sequences were extracted from the reference using bedtools getFasta (Quinlan & Hal, 2010). Initial bait QC found that sequences had high GC content on average, so we opted to design two 80 bp baits offset by 20bp for each variable locus. These sequences were input into Arbor Biosciences’ complementary bait design pipeline. In order to maintain a bait, we required 0 off‐target hits with a *T*_m_ > 60°C, fewer than 10 off‐target hits with Tm between 62.5 and 65°C, and at most 1 off‐target hits with *T*_m_ > 65°C. We also required 0 alignments to the sea lamprey mitome, dG >= −15, 0 heterodimers with other baits, and less than 25% repeat masking. Two 80 bp sequences were selected for each variable RAD locus (*n* = 3,764 loci).

RAD capture libraries were prepared using the library preparation protocol described above for the discovery libraries; however, libraries were sheared using the default protocol for 300 base pair fragments using a Covaris M220 Ultrasonicator (Covaris), and following PCR, equal amounts of each library (50 ng) were pooled before proceeding with a MyBaits hybridization capture reaction. Adult samples from Brule River, Carp River, and St. Mary's River were also included in these libraries. The capture reaction was carried out using the MyBaits Version 4.01 protocol (https://arborbiosci.com/wp-content/uploads/2018/04/myBaits-Manual-v4.pdf; April 2018). RNA baits were allowed to hybridize to sea lamprey genomic target sites for 16 hr at 65°C and then washed with Wash Buffer X at between 65 and 67°C. Washed capture reactions were amplified for 11 cycles using KAPA library amplification kit for Illumina (KAPA Biosystems) and quantified using three replicate PicoGreen assays. We determined that the library was free of adapter dimers and was of the correct insert size by evaluating the electropherogram produced by a Bioanalyzer High Sensitivity DNA Assay (Agilent Technologies). The library was again quantified and checked for adapter dimers using qPCR and a Bioanalyzer assays, respectively, before being sequenced in a single HiSeq X lane (Illumina) using a 5% phiX spike‐in (Novogene Corporation).

### 2.4 RAPTURE bioinformatics

Quality of RAPTURE sequencing data was initially inspected using FastQC (Andrews, 2014). Reads in the two files were exchanged whenever the barcode was found at the start of read 2 using a custom perl script (bRAD_flip_trim.pl), reads were demultiplexed using process_radtags (‐‐inline_null ‐e sbfI ‐‐barcode_dist_1 1 ‐‐retain_header), and PCR duplicates were removed using clone_filter (Catchen et al., 2011). Adapter sequences were removed from the end of reads using Trimmomatic, and reads were trimmed whenever the mean base quality across a sliding window of four bases dropped below Q15 (Bolger, Lohse, & Usadel, 2014). Read pairs were removed from the dataset if one or both reads were less than 50 bp after trimming. To create a reference to map remaining reads to, we merged the sea lamprey reference genome (Smith et al., 2018; GenBank: PIZI00000000.1) and mitome (Lee & Kocher, 1995; GenBank: U11880.1), resulting in a fasta file that was normalized using Picard NormalizeFasta (http://broadinstitute.github.io/picard). Read mapping was performed using BWA‐mem with default settings (Li, 2013). Mapped bam files were filtered using SAMtools view in order to exclude reads with mapping qualities <30 and reads not mapping in proper pairs. SAMtools was also used to sort and index filtered bam files (Li et al., 2009).

Variants were detected, and genotypes were called using gstacks (Catchen et al., 2011; Maruki & Lynch, 2017) and FreeBayes (Garrison & Marth, 2012). Gstacks was run using default parameters, and FreeBayes was run without applying population or binomial observation priors, an expected cross‐contamination rate of 1%, and a minimum base quality score of 20. Variants were only reported whether the alternate allele was supported by greater than 2 reads and whether the total depth across all individuals was greater than 1000XBiallelic SNPs that were detected by both programs and overlapped targeted RAD loci were extracted from the FreeBayes VCF file using VCFtools (Danecek et al., 2011). VCFtools was also used to remove individuals with greater than 75% missing data and set genotypes to missing whether the genotype quality (GQ) was less than 10. We also required genotypes at a site to have a minimum mean depth of 10× and no more than 30% missing genotypes. Sequencing characteristics of the RAPTURE panel, including allele read balance and mean total depth for each variable position, are reported as part of an R Markdown document on the project's GitHub repository.

### 2.5 Estimation of population diversity and differentiation using RAD capture panel

Summary statistics describing levels of inter‐ and intrapopulation diversity were calculated using genotypes called by FreeBayes from the RAD capture dataset. Before calculating summary statistics, DC samples were reduced to include only a single individual from each full‐sibling family group based on pedigree reconstructions described below. No SCR larvae pairs were full siblings so all individuals were used. Individuals with greater than 50% missing data were also removed at this point, leaving 140 individuals for analyses.

When attempting to establish pedigree relationships, population measures of genetic diversity are important to the accuracy of inferred interindividual relationships (Blouin, 2003). Specifically, biallelic SNPs with high minor allele frequencies are expected to be most informative for determining kinship (Krawczak, 1999). Genetic diversity measures including observed heterozygosity (*H*_O_), expected heterozygosity (*H*_E_), Wright's inbreeding coefficient (*F*_IS_), and minor allele frequencies (MAFs) were calculated for each locus–population combination. MAFs were calculated using the “minorAllele” function of the R package adegenet (Jombart, 2008; Jombart & Ahmed, 2011), and *H*_O_, *H*_E_, and *F*_IS_ values were calculated using the “basic.stats” function of the R package hierfstat (Goudet, 2004). Distributions of MAF and *F*_IS_ were plotted using the R package ggplot2 (Wickham, 2016). RAD loci were plotted on scaffolds using the R function “preparegenome plot” from the quantsmooth package (Oosting, Eilers, & Menezes, 2018).

Given that the SNP discovery library was constructed from individuals sampled from two populations, we were interested to document whether the SNP loci were polymorphic in other Great Lake tributaries and basins, and to document levels of interpopulation variance in allele frequency. Pairwise estimates of *F*_ST_ (Nei, 1973) were estimated among sampling sites using the “pairwise_Gst_Nei” and global *F*_ST_ was estimated using “diff_stats,” both from the mmod R package (Winter, 2012). Differentiation statistics were estimated using the 140 individual downsampled dataset (Table 1).

Discriminant analysis of principal components (DAPC; Jombart, Devillard, & Balloux, 2010) was used to examine patterns of interpopulation spatial genetic structure. DAPC provides a multivariate clustering approach that summarizes genetic data into a user‐defined number of principal components before performing discriminant analysis on those retained principal components. This strategy maximizes variation among groups while minimizing variance within groups, providing a means of detecting genetic structure (Jombart et al., 2010). Contributions of individual SNP loci to the DAPC can be used to determine which loci most strongly influence the observed patterns (Jombart et al., 2010).Locus loadings have different ranges along each axis and were therefore transformed and presented as percentile ranks to allow for comparisons. DAPC was performed using the adegenet R package using the downsampled dataset.

### 2.6 Detecting outlier loci

Genotypes for thousands of SNPs allowed analyses to ascertain whether some loci appear to be acting in a non‐neutral manner and/or explain a disproportional amount of variation among population samples. We used the R package OutFLANK (Whitlock & Lotterhos, 2015) to detect loci potentially experiencing selection. OutFLANK uses a trimmed distribution of locus‐specific *F*_ST_ values to infer a distribution of neutral markers. Loci with significant deviations based on this expected distribution are considered outliers and possibly subject to selection. We used the default values of trimming 5% of loci from each tail of the overall *F*_ST_ distribution and a 0.10 false discovery rate (Benjamini & Hochberg, 1995) to reduce the likelihood of false‐positive detections. Outlier analyses were also conducted using the dataset that was downsampled to include a single individual from each full‐sibling family.

### 2.7 Assessment of power for larval sea lamprey pedigree reconstruction

The power to correctly infer full‐ and half‐sibling relationships among larval offspring when no adult lamprey were genotyped was assessed using simulated pedigrees analyzed using the genetic pedigree reconstruction assignment program COLONY (Jones & Wang, 2010; Wang, 2004). Each simulation began by creating a breeding matrix where the total number of parents (*N*_parents_) was assumed to be 10, 100, or 1,000. These numbers were chosen to assess assignment power over a broad range of possible breeding scenarios and spawning adult abundance. For each simulation, the size of the breeding matrix (*N*_females_ × *N*_males_) was allowed to vary such that the *N*_males_:*N*_females_ sex ratio was sampled from a uniform distribution (range: 1–2, Hansen et al., 2016). For *i* in 1 to *N*_females_ columns in the matrix, the number of males (*N*_mates_) that mated with the *i*th female was randomly drawn from a Poisson distribution (*λ* = 3), given that evidence for polygyny and polyandry was established by Gilmore (2004). The number of eggs fertilized (*N*_offspring_) for the *i*th female was sampled from a uniform distribution (range: 25,000–1,00,000, Manion & Hanson, 1980). For each female, *N*_offspring_ were nonrandomly distributed among mated males following Equation (1). The random process of identifying mates per female sometimes resulted in a limited number of males not mating with any females. To ensure the sex ratio remained relatively unaltered, we repeated the above process with any unmated males, except that *N*_mates_ were females rather than males.

(1) $$N_{\text{offspring}}\times \frac{N_{\text{mates}},N_{\text{mates}}-1,\ldots ,1}{\text{sum}\left(1,2,\ldots ,N_{\text{mates}}\right)}$$

Thus, each simulation creates a polygamous mating structure, not just a polyandrous mating structure because multiple matings, from the perspective of males or females, occurred by chance. As noted above, the number of mates was drawn from a Poisson distribution, but the actual individuals mated were chosen randomly. The mean number of mates per sex was driven by the sex ratio (ranges from 1 to 2 males per female, drawn randomly) and the size of the breeding matrix (containing 10, 100, or 1,000 total adults). In the simulations, the average number of mates for a female was ~3 (range 2.7–3.2 across breeding matrices simulated) and for a male, it was ~2 (range: 1.5–2.2 across breeding matrices simulated).

After the breeding matrix was created, 100 offspring were randomly sampled from the total number produced by the breeding matrix. Each set of *N*_parents_ simulations was evaluated at 100, 200, or 500 independent loci (*N*_loci_), where the allele frequencies for *N*_loci_ were randomly drawn from the observed allele frequencies based on the original RAD discovery dataset. The above information was provided to the simulation module within COLONY (Wang, 2013) using in‐house wrapper scripts in R (version 3.5, R Core Team, 2018) to generate 100 independent simulated datasets to be run in COLONY (available in the GitHub repository). Thus, a total of 900 independent simulated datasets were evaluated across the nine different combinations of *N*_parents_ and *N*_loci_.

When analyzing the simulated datasets in COLONY, we assumed a polygamous mating system and no sibship prior was used. Each genetic pedigree was inferred using the full‐likelihood method, with high precision and a “long” run. We used the Linux version of COLONY (colony2s.ifort.out, version 2.06) to infer full‐ and half‐sibling relationships for each independent simulation. Analyses were conducted in parallel on MSU iCER HPCC. For each simulation, the “Best Configuration” genetic pedigree was analyzed to assess power. Importantly, known parental genotypes were not provided to COLONY when the program was reconstructing the pedigrees, but that the parents that actually produced any given offspring were known, as their names were embedded in the names of their respective offspring. In addition, these data require comparisons between inferred and known dyadic (pairwise) relationships to assess error and accuracy (below). That is, the inferred (via COLONY) and known (based on simulation information embedded in offspring names) familial relationship was determined by counting how many parents the two individuals shared: Full siblings share both parents, half‐siblings share one parent, and unrelated dyads share no parents. Using this information, accuracy, the false‐positive rate and the false‐negative rate can be calculated for each simulation. Here, accuracy is defined as the proportion of inferred dyads (full‐sibling, half‐sibling, or unrelated dyads) that were correctly assigned. The false‐positive rate is 1‐accuracy (i.e., the proportion of inferred dyads (full‐sibling, half‐sibling, or unrelated dyads) that were incorrectly assigned). Given the direct relationship between accuracy and the false‐positive rate, we report only accuracy. The false‐negative rate is defined as the proportion of known dyads (full‐sibling, half‐sibling, or unrelated dyads) that were incorrectly inferred. The expected distributions of accuracy and false‐negative rates for full‐sibling, half‐sibling, and unrelated dyads across 10, 100, or 1,000 parents genotyped at 100, 200, or 500 loci are presented below. Note that COLONY does provide probabilities that any given dyad in the genetic pedigree reconstructed is true, but no user‐defined cutoff was chosen because we implemented the full‐likelihood method of COLONY, which effectively balances the false‐positive and false‐negative rates throughout the dataset (Jones & Wang, 2010; Wang, 2004). Additional details, including step‐by‐step instructions, are available in the project's GitHub repository.

### 2.8 Pedigree reconstruction

We inferred full‐ and half‐sibling relationships for each age cohort of larval sea lampreys sampled within DC and the SCR separately in COLONY. Larval age was estimated using length–frequency distributions of larvae sampled in each stream (as described above). The above simulations assumed that loci were independent of each other. Thus, we selected loci for genetic pedigree reconstruction that were two megabases apart on the same chromosome (i.e., independent, as determined from Smith et al., 2018) because the RAPTURE panel contained multiple loci per chromosome (see below). Loci chosen were genotyped in >80% of individuals (97% ± 3%, mean ± 1*SD*) and had high minor allele frequencies (0.20 ± 0.20, median: 0.12). The number of loci per chromosome varied based on chromosome size (range: 1–14, median = 1). The above filtering identified 454 independent loci to be used in genetic pedigree reconstruction, which is comparable to the simulations based on 500 loci (see above). All other COLONY input parameters were the same as described for the simulations above.

We also estimated the effective number of breeders (*N*_b_) that produced each larval sample using the sibship method based on pedigree membership to full‐ and half‐sibling groups, as implemented in program COLONY (Wang, 2004, 2009). *N*_b_ is typically lower that the number of adults associated with pedigreed offspring because the variance in offspring produced by adults and realized breeding sex ratio (Araki, Waples, Ardren, Cooper, & Blouin, 2007; Duong, Scribner, Forsythe, Crossman, & Baker, 2013). We also estimated the effective number of breeders (*N*_b_) using the LDNe method (Waples & Do, 2008) as implemented in NeEstimator Version 2.1 (Do et al., 2014). We used the random mating model, a *P*_crit_ allele frequency cutoff of 0.05, and only considered interscaffold locus pairs. Reported confidence intervals were generated by jackknifing across samples. We also repeated the analysis using all locus pairs in order to determine the extent to which including physically linked loci would downwardly bias *N*_b_ estimates.

### 2.9 Statistical analyses for pedigrees

Assuming directional (downstream) dispersal of offspring and assuming the farthest upstream locale where a full or half‐sibling was collected were a proxy for the spawning location, we were able to coarsely characterize larval dispersal. Randomization procedures were used to test whether the observed number of within sampling location dyads that were related (*N*_RW_) was due to chance alone. The randomization procedure first enumerated all possible pairwise relationships among the number of genotyped (*N*_gt_) offspring (i.e., ${\text{Dyad}}_{\text{total}}=N_{\text{gt}}!/\left(2!\left(N_{\text{gt}}-2\right)!\right)$), with individual sampling locations recorded, as well. *N*_RW_ dyads were randomly assigned as related, and the proportion of N_RW_ observed within the same sampling locations, $\left(P_{\text{NW}}=N_{\text{RW}}/{\text{Dyad}}_{\text{total}}\right)$, was recorded. The above procedure was repeated 1,000 times to generate a null distribution, representing the expected proportion of dyads observed within sampling locations due to chance. We calculated statistical significance as the proportion of the null distribution greater than the observed *P*_NW_ per cohort. In addition, Fisher's exact tests were used to test whether the *P*_NW_ per cohort differed significantly and thus whether spatial dispersion of larvae was concordant among age cohorts. Finally, a measure of coancestry was calculated per pedigree using an R function created by Bartron, Sard, and Scribner (2018), which was based on Cockerham's (1967) derivation of coancestry for a group of individuals. All statistical tests were conducted at *α* = 0.05. All analyses and graphics were completed in R, aided by “tidyverse” (Wickham, 2017).

## 3 RESULTS

### 3.1 Genetic verification of species identity

Species identity of putative sea lamprey larvae for the SNP discovery panel was validated based on amplification using the sea lamprey specific mitochondrial COI PCR primers (Gingera et al., 2016). All larvae returned to the laboratory and screened from DC and SCR were sea lampreys based on positive PCR amplification.

### 3.2 Sequencing results and genotyping

In our discovery library, 319,995,167 2 × 150 reads were obtained from the sequencing run. The mean PCR duplication rate among individuals was 13.2%. Following moving barcodes to read‐1 and demultiplexing samples, 225,992,151 reads remained for the identification of variable RAD loci and assembly of consensus sequences needed for bait design. After filtering, we retained bait pairs for 3,764 variable RAD loci.

The RAPTURE library obtained a total of 380,610,906 2 × 150 paired‐end reads from the sequencing run. A total of 271,842,286 demultiplexed read pairs were obtained across all individuals (71.4%), with an average of 688,208 reads per individual. The mean PCR duplication rate was 50.7%, and we retained a total of 133,265,326 reads after removing duplicates. On average, 93.1% of read pairs were maintained after quality trimming and removing adapter contamination. We retained a total of 123,325,992 read pairs after trimming. A total of 110,472,816 read pairs were mapped to the sea lamprey reference genome in proper pairs with mapping qualities (MQ) greater than 30.

FreeBayes detected a total of 33,341 variable SNPs and indels overlapping the coordinates of targeted RAD loci. Of these loci, 8,540 were indels and 24,801 were SNPs. Of these SNPs, 23,950 had two alleles and 851 had more than 2 alleles (including the reference allele). Gstacks detected and called 71,705 biallelic SNPs that overlapped the coordinates of targeted RAD loci. A total of 19,510 biallelic SNPs were identified by both genotyping algorithms. Only SNPs occurring in both methods were retained for further analysis. We retained 12,435 SNPs after filtering the VCF generated by FreeBayes. After downsampling individuals from DC to include only one individual from each full‐sibling family and removing individuals with >50% missing data, we were left with 11,818 biallelic SNPs and 140 individuals. Sequences for baits used for genotyping the analyzed SNPs are available on GitHub (http://github.com/ScribnerLab/SeaLampreyRapture).

Coordinates for recovered targeted RAD loci were extracted from the fasta file produced by gstacks and cross‐referenced with mapping coordinates for targeted loci using bedtools intersect (Quinlan & Hal, 2010). We recovered sequencing data for 3,446 of 3,764 (91.6%) targeted RAD loci. The small disparity between the number of baited loci and the number of recovered loci could be caused by several factors. In this case, removing reads with MQ < 30 before processing RAPTURE data likely led to the exclusion of baited RAD loci from low complexity regions of the genome with systematically low mapping qualities.

**Figure 2:**
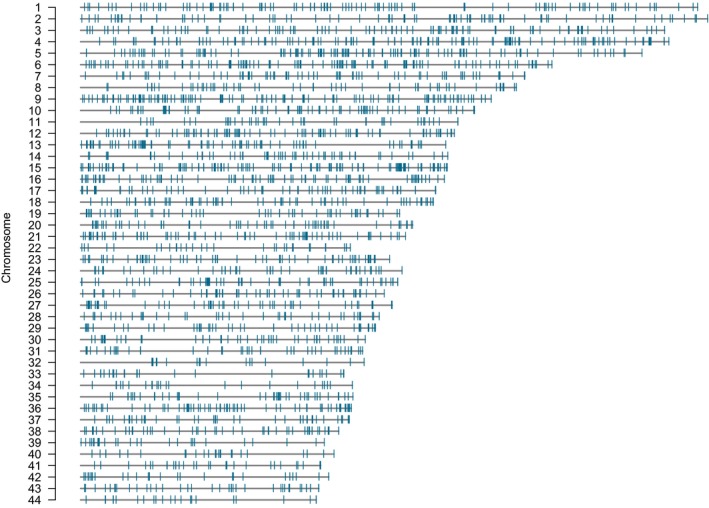
Distribution of target RAD loci across the 44 largest scaffolds (>10 MB) in the sea lamprey genome. Each horizontal black line represents a scaffold, and each vertical blue line represents the location of a targeted locus. A total of 2,844 of 3,446 (82.5%) targeted loci map to these scaffolds

A total of 80.6% of reads aligned to recovered target RAD tags. The mean density of targeted loci across scaffolds > 10 megabases (MB) in length was 4.02 RAD loci (*SD* = 1.30) per megabase. Targeted loci aligned to 188 out of 12,061 scaffolds from the Smith et al. (2018) germline genome assembly, with 2,844 out of 3,446 RAD loci (82.5%) aligning to scaffolds greater than 10 MB in length (*n* = 44; Figure 2) and 3,303 loci (95.85%) aligning to scaffolds larger than 1 MB. Allele read balance (AB) measurements were obtained for all 12,435 SNPs. Allele read balance (AB) was near the expected value (0.5) on average suggesting that genotype calling was not confounded by sequencing or mapping errors. AB values for our dataset ranged from 0.071 to 0.888, with a mean value of 0.478 and a standard deviation of 0.07. That is, the vast majority (96.2%) of loci yielded AB values between 0.3 and 0.7 (*n* = 11,377). Previous studies have found that SNP loci with AB values between 0.3 and 0.7 can often be validated using Sanger sequencing (Krumm et al., 2015). A comprehensive characterization of the RAPTURE sequencing output is available at github.com/ScribnerLab/SeaLampreyRapture.

### 3.3 Genetic diversity and differentiation

Locus‐ and population‐specific measures of genetic diversity including observed heterozygosity (*H*_o_), expected heterozygosity (*H*_e_), degree of deviation of observed from expected heterozygosity (*F*_IS_), and minor allele frequency (MAF) are provided in the GitHub repository. Distributions of *F*_IS_ were slightly positive in all populations examined, with Duffins Creek having the largest observed deficit of heterozygotes (*F*_IS_ means: DC = 0.0118, SCR = 0.045, St. Mary's River = 0.043, Brule River = 0.018, Carp = 0.035; Table 2). The distribution of MAF varied among populations sampled, with Duffins Creek having the highest average minor allele frequency (means: DC = 0.168, SCR = 0.157, St. Mary's = 0.156, Brule = 0.155, Carp River = 0.154).

**Table 2:** Measures of genetic diversity for five sea lamprey spawning sites, including mean observed heterozygosity (*H*_o_), mean expected heterozygosity (*H*_e_), Wright's inbreeding coefficient (*F*_IS_), and mean minor allele frequency (MAF)

Sampling site	*H*_o_	*H*_e_	*F*_IS_	MAF
Brule River	0.215	0.219	0.018	0.155
Carp River	0.213	0.222	0.035	0.154
Duffins Creek	0.214	0.245	0.118	0.168
St. Clair River	0.212	0.223	0.045	0.157
St. Mary's River	0.211	0.222	0.043	0.156

### 3.4 Estimates of spatial genetic structure

Locus‐specific F_ST_ estimating interpopulation variance in SNP allele frequency among five Great Lakes tributary samples ranged from −0.019 to 0.233 (mean = 0.011). Estimates were obtained for all 11,818 SNPs that were retained after sample sizes were reduced for the DC population. Global interpopulation *F*_ST_ across all loci and populations was 0.01, and mean pairwise *F*_ST_ values between populations ranged from −0.001 to 0.0018 (Table 3). Pairwise comparisons indicated the strongest differentiation existed between DC (Lake Ontario basin) and the upper Great Lakes sampling sites (Table 3).

**Table 3:** Pairwise *F*_ST_ estimates for sea lamprey sampled from five spawning streams, including age‐specific variation among cohorts (1, 2, 3 refer to age classes) sampled from Duffins Creek (DC) and the St. Clair River (SCR)

	BR	CARP	DC‐1	DC‐2	DC‐3	SC‐2	SC‐3	SM
BR	—							
CARP	0.002	—						
DC‐1	0.013	0.011	—					
DC‐2	0.014	0.012	0.004	—				
DC‐3	0.018	0.015	0.008	−0.001	—			
SC‐2	0.001	0.001	0.010	0.011	0.015	—		
SC‐3	0.002	0.000	0.012	0.012	0.016	0.000	—	
SM	0.001	0.001	0.011	0.011	0.016	0.000	0.001	—

Abbreviations: BR, Brule River (Lake Superior); CARP, Carp River (Lake Michigan); DC, Duffins Creek (Lake Ontario); SCR, St. Clair River; SM, St. Mary's (Lake Huron).

**Figure 3:**
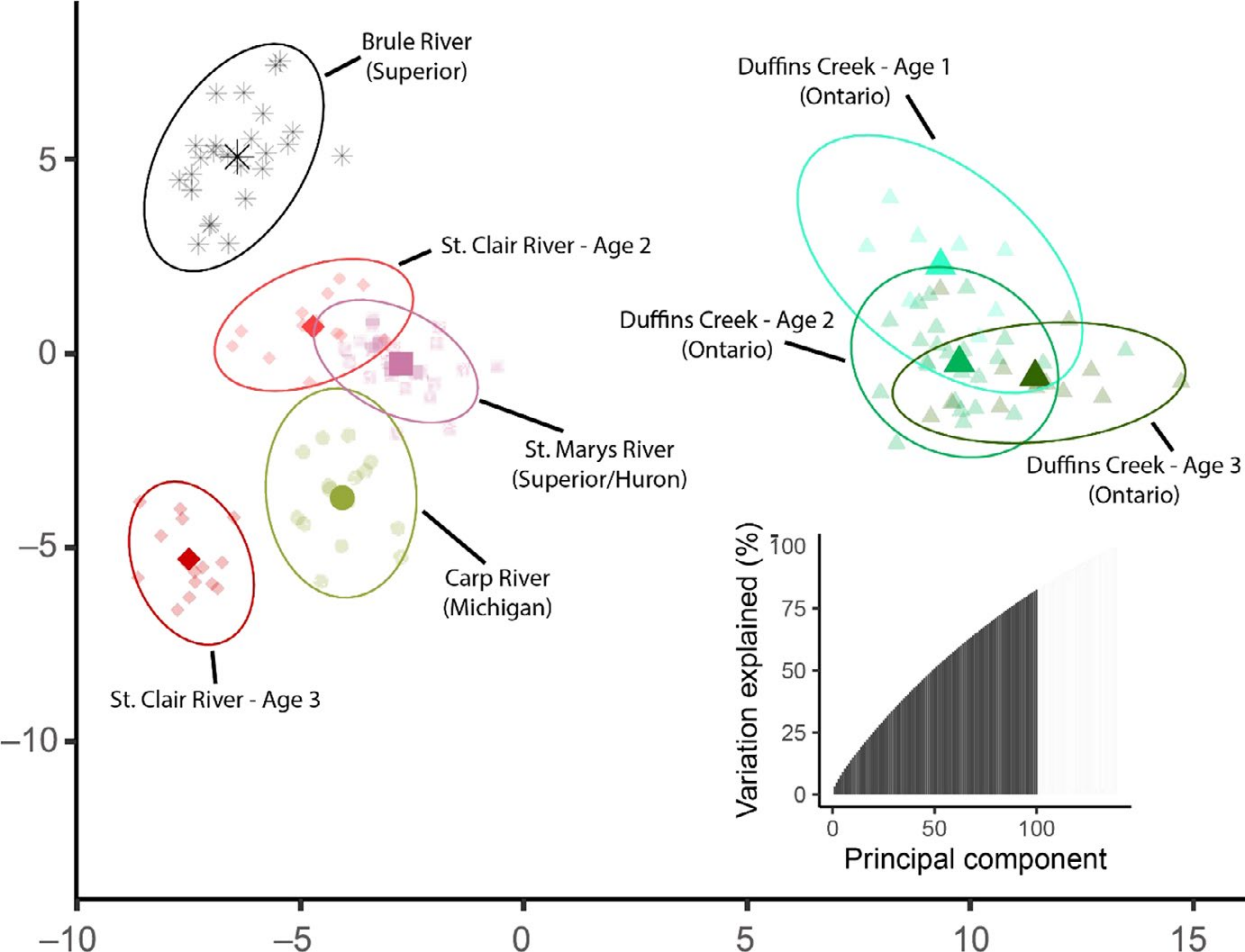
Ordination of discriminant analysis of principal components (DAPC) for individual larval sea lamprey sampled from five spawning streams, including multiple cohorts from Duffins Creek and the St. Clair River. 95% confidence ellipses are also provided for each sampling site. The inset figure illustrates cumulative percent of variation explained for 100 retained principal components

Using DAPC, we transformed SNP variation over all loci into 149 principal components (PCs), of which we retained 100 based on Jombart et al. (2010). Retained PCs captured 82.5% of among sample genomic variation. The first retained linear discriminant axis separated DC (Lake Ontario basin) from the upper Great Lakes sampling sites, particularly Brule River from the western Lake Superior basin. The second axis provided south to north discernment within the set of upper Great Lakes sites (Figure 3).

**Figure 4:**
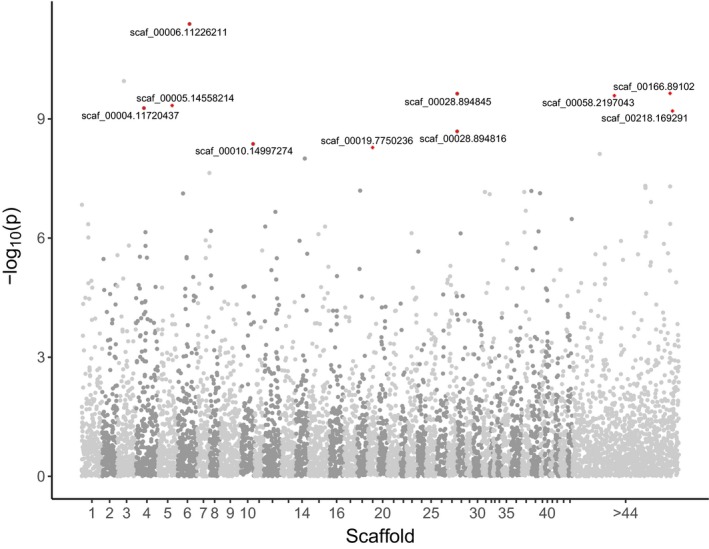
Manhattan plot showing distributions of ‐log_10_‐transformed *p*‐values associated with interpopulation *F*_ST_ for genotyped SNPs. Outlier loci detected using OutFLANK are labeled and shown in red along with scaffold and position (bp)

We identified 10 loci that significantly deviated from the expected *F*_ST_ distribution for neutral loci. Manhattan plots of distributions of –log_10_‐transformed *p*‐values (Figure 4) show that outlier loci are distributed across nine scaffolds. Levels of *F*_ST_ estimated by OutFLANK for these loci ranged from 0.116 to 0.233.

In some cases, outlier loci contributed disproportionally to the ordination of samples from different populations and cohorts in multivariate space based on DAPC. Specifically, results suggest that these loci may contribute to differentiation among individuals from DC (Lake Ontario) and the remainder of the sampling locations (discriminant function 1 axis) and among samples from other Great Lakes tributary locations (discriminant function 2 axis). Because of the species' semelparous life history, individuals from different cohorts are unlikely to be related (full siblings or half‐siblings), and the multivariate ordination of individuals from the age‐2 and age‐3 larval cohorts from the SCR cluster is interesting. Cohorts cluster separately and with adults from different populations, suggesting different origins of individuals spawning in the St. Clair River in different years (Figure 3).

### 3.5 Assessment of power of larval sea lamprey pedigree assignment

**Figure 5:**
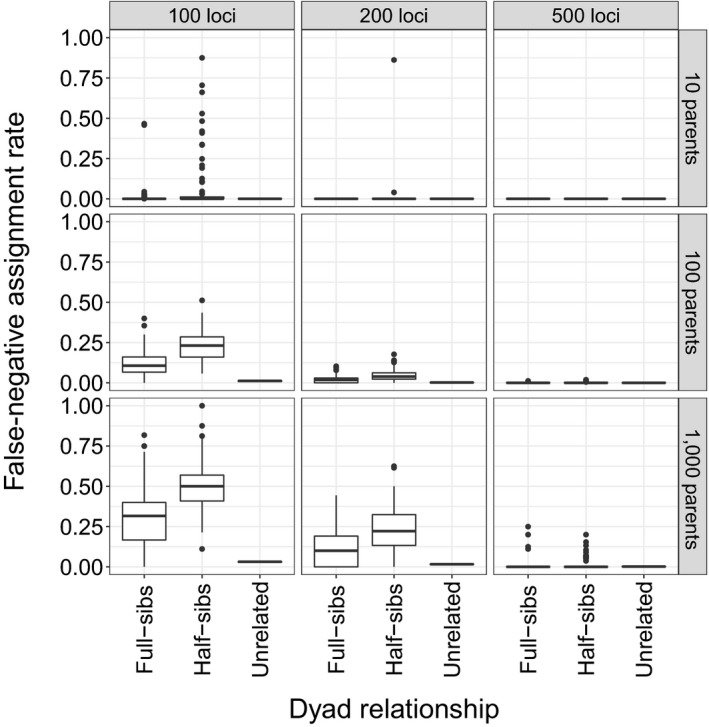
Boxplot distributions of the proportion of known full‐sibling, half‐sibling, and unrelated dyads that were incorrectly (i.e., false‐negative rate) inferred across 10, 100, or 1,000 parents genotyped at 100, 200, or 500 loci

Across all simulations, the power to correctly infer known related dyads increased when more loci were used (Figure 5). Conversely, resolving sibling relationships became more difficult when more parents were used in the breeding matrix. Initially, we evaluated false‐negative rate (proportion of known dyads incorrectly inferred) across all *N*_parents_ × *N*_loci_ comparisons. Generally, most true full‐ and half‐sibling dyads were identified using 200 or 500 loci when 10, 100, or 1,000 parents contributed to simulated offspring (Figure 5). Across all parameters evaluated, most unrelated dyads could be resolved as well (Figure 5).

**Figure 6:**
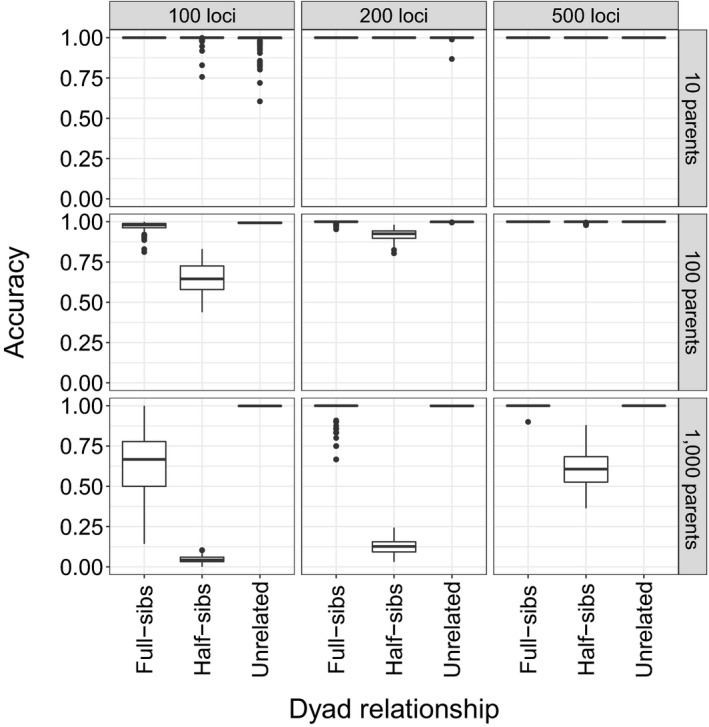
Boxplot distributions of the proportion of inferred full‐sibling, half‐sibling, and unrelated dyads that were correct (i.e., accuracy) across 10, 100, or 1,000 parents genotyped at 100, 200, or 500 loci

Given that interindividual dyadic relationships will not be known in datasets generated by field sampling and subsequent genotyping, it was important to assess accuracy among inferred dyads (the proportion of inferred dyads that were correctly inferred). Most inferred full‐sibling and unrelated dyads were correctly inferred across most *N*_parents_ × *N*_loci_ comparisons (Figure 6). Accuracy was related to both *N*_parent_ and *N*_loci_. More half‐sibling dyads were incorrectly inferred when *N*_parents_ increased from 10 to 1,000. However, increasing *N*_loci_ (100 to 500) resulted in a great improvement in assignment accuracy.

### 3.6 Larval sea lamprey pedigree assignments in the SCR and DC

A total of 250 larval lampreys were successfully genotyped from DC (*n* = 217) and the SCR (*n* = 33). Parameters estimated for cohorts from DC are likely tied to larval sample sizes that varied across age classes (Table 1). The number of independent adult genotypes inferred for each genetic pedigree in DC varied among cohorts (*N*_s_ range: 16–42). The mean and variance in adult reproductive success varied across cohorts (Table 4). High variance in reproductive success for age‐1 and age‐2 larvae is reflected in comparatively lower estimated effective numbers of breeding adults (*N*_b_) in age‐1 and age‐2 age cohorts relative to the age‐3 cohort (Table 4). Mean coancestry ranged from 0.03 to 0.10 across the three cohorts sampled in DC (Table 4), largely reflecting differences in numbers of breeding adults, adult mean and variance in reproductive success, and number of full‐ and half‐sibling dyads (Table 3). Estimates of *N*_b_ based on the number and variability in family size within cohorts (Wang, 2004 estimator) were comparable to those based on linkage disequilibrium (Table 4). Following genetic pedigree reconstruction in COLONY, no related offspring were inferred in either of the SCR cohorts; therefore, the number of adults estimated to have produce 33 offspring was 66.

**Table 4:** Summary statistics from pedigree analyses for larval sea lamprey of different age classes collected from two streams

Statistic	Duffins Creek age cohorts			St. Clair River age cohorts	
	1	2	3+	2	3+
No. of larvae sampled	38	146	30	15	16
Mean (variance) in adult RS^a^	4.8 (58.3)	7.0 (149.2)	2.4 (5.2)	NA	NA
N_b_ (95% CI)^b^	5 (2–20)	8 (4–24)	17 (10–35)	NA	NA
N_b_ (95% CI)^c^	6 (3–10)	10 (8–12)	14 (9–24)	NA	NA
N_b_ (95% CI)^d^	6 (3–10)	10 (8–12)	14 (9–24)	NA	NA
Obs. Ns^e^	16	42	25	30	32
No. of full‐sib dyads	568	2,926	84	0	0
No. of half‐sib dyads	24	2002	40	0	0
Cohort coancestry	0.103	0.046	0.030	0	0
Cohort relatedness	0.26	0.13	0.06	NA	NA

a Reproductive success defined as the number of offspring assigned to unsampled parents based on pedigree reconstruction.

b Effective number of breeding adults (Wang, 2004) producing larvae for each cohort and stream.

c Effective number of breeding adults (Waples & Do, 2008) estimated based on linkage disequilibrium; only interscaffold locus pairs included.

d Effective number of breeding adults (Waples & Do, 2008) estimated based on linkage disequilibrium; all locus pairs were included.

e Number of adults producing larvae in inferred pedigrees for each age class and stream.

**Figure 7:**
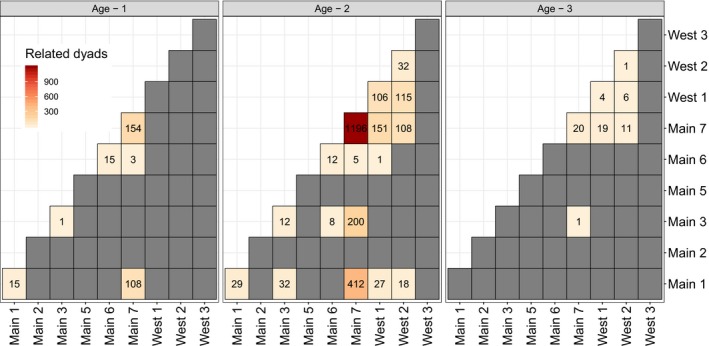
Heatmaps representing related dyadic relationships observed for age‐1 (*n* = 38), age‐2 (*n* = 146), and age‐3 (*n* = 30) larval sea lamprey among sampling locations in Duffins Creek (Figure 1). Darker red colors indicate more related dyads observed. Actual dyads observed per pairwise (location) comparison are reported within corresponding cells. Gray cells represent no dyads observed. Note cells along the diagonal represent dyadic relationships within sampling location and that location “Main 4” was not included because none collected from that location (*n* = 3) were successfully genotyped

Clusters of related (full and half‐sibling) individuals were observed in DC for each cohort (details in GitHub repository). Randomization tests sought to determine whether the pattern of related individuals within sampling locales was the result of sampling locations for specific larvae. Results indicated that related larval lamprey were not randomly dispersed in DC (i.e., not the product of variation in larval sample sizes across sampling locations). Based on the broad spatial scale over which larval samples were collected (Figure 1), adult lamprey appear to have successfully bred in a limited number of locations. Randomizations were not conducted with SCR larvae because no related dyads (either full or half‐siblings) were inferred. Age‐1 and age‐2 cohorts evaluated in DC contained clusters of larvae in specific sampling locales that were not expected by chance (*p* < .001). The most upstream sampling location in DC (Main 7; Figure 1) consistently contained the largest number of related dyads in all sampled cohorts (Figure 7), suggesting this section of stream was consistently used for spawning. There was evidence of downstream dispersal based on observations of co‐occurrence of full and half‐siblings in multiple stream collection sites (range: 1–1,196 dyads) in each cohort (Figure 7). Importantly, some individual sea lampreys spawn in very different locations based on dispersion of larvae. Most related dyads where individuals were sampled in different locations were observed in the main stem of DC in two of the three cohorts evaluated (Figure 7). The sampling location (Main 1) with the fewest samples (below the confluence of the main stem and western branch of DC) consistently contained larvae related to other larvae collected upstream (Figure 7). The spatial distribution of dyads (*P*_NW_ per section pair) differed significantly (*p* < .05) among age‐1, age‐2, and age‐3 and older cohorts sampled in DC.

## 4 DISCUSSION

The project produced a large suite of SNPs (*N* = 11,818), and a rapid and a cost‐effective assay to facilitate future sea lamprey genotyping studies. We demonstrated applications of SNP loci associated with objectives to quantify aspects of sea lamprey reproductive ecology including estimation of the number of adults associated with larvae based on pedigree analysis. Simulations accompanying pedigree analyses of larval sea lampreys collected demonstrated high accuracy of pedigree assignment even in situations of high adult abundance. Results from sampling just over 200 larvae from DC (a) are consistent with previous studies that indicate sea lamprey display a polygamous mating behavior (Gilmore, 2004; Rodrıguez‐Munoz & Tregenza, 2009), and (b) showed that downstream dispersal is a continuous process for individuals of different ages (Derosier et al., 2007). Population genetic analyses demonstrate that the SNPs developed from larval sea lampreys from two populations were variable across the Great Lakes and exhibit levels of interpopulation variance in allele frequency that are comparable to mtDNA (Waldman et al., 2004) and microsatellite analyses (Bryan et al., 2005).Identification of outlier loci (Figure 4; features detailed in GitHub repository) portends developments in areas of gene‐assisted control (McCauley et al., 2015), for example, the exploration of potential rapid evolution in response to intense selection associated with pesticide treatments (Dunlop et al., 2018).

### 4.1 Evidence for spatial genetic structure

Our genetic structure results are consistent with previous analyses of spatial genetic structure of sea lampreys across the Great Lakes (Bryan et al., 2005; Waldman et al., 2004) based on microsatellites. This result was thus expected because sea lampreys do not home to natal streams. Little evidence of genetic differentiation was documented among populations within each Great Lake basin, consistent with tagging studies (Bergstedt & Seelye, 1995) that have demonstrated lack of natal homing. Data on SNP allele frequency variation within Great Lakes basins relative to more substantial variation among basins are consistent with the hypothesis that adult sea lampreys select streams based on olfactory queues associated with larval abundance in streams rather than cues associated with natal origins (Buchinger, Siefkes, Zielinski, Brant, & Li, 2015).Previous analyses of sea lamprey spatial genetic structure in terms of genetic affinities among populations based on mtDNA (Waldman et al., 2004) and microsatellite loci (Bryan et al., 2005), and coalescence‐based model evaluation and estimation of levels of population bottlenecks (Bryan et al., 2005) indicated a sequential pattern of movements from Lake Ontario to Lake Erie and then into the upper Great Lakes.

### 4.2 Effects of outlier loci on spatial population structure

Information concerning interpopulation variance in SNP allele frequency could inform future applications of these markers in areas of sea lamprey control or conservation. Emerging DNA methods are being widely used to detect environmentally mediated selection that can enhance the ability of an invasive species to thrive in introduced habitats (Ellner & Sasaki, 1996). Populations diverge genetically as a function of the length of time they have been isolated, levels of gene flow, and based on genotypic (or genome‐level) responses to selection mediated by environmental features (Garcia de Leaniz et al., 2007) that likely vary among lake and tributary locations within and across lake basins. Accordingly, genetic data can provide important information concerning processes of colonization, dispersal, and adaptive response environments by invasive species from areas of introduction. Spatial variation in environmental conditions can exert selective pressures that lead to different abilities to survive and reproduce. Analyses of thousands of loci mapped across the genome make possible the identification of loci that might be under selection, associated with environmental variables, and potentially adaptive (Allendorf et al., 2010). For example, analyses can identify “outlier” loci that show unusually high or low population differentiation (Oleksyk, Smith, & O'Brien, 2010). Such outlier loci and environmentally associated variation in the frequency of alleles at these loci can help identify potentially adaptive genes and source habitats of origin.

Outlier loci (*n* = 10 of 11,818 loci) were documented based on differences in SNP allele frequency across populations. Interestingly, outlier loci appear to contribute disproportionately to loadings on the DAPC axis that separates DC from all other populations evaluated (Figure 3). The presence of outlier loci (Figure 4) with *F*_ST_ values orders of magnitude higher than the mean suggest that during the process of colonization of the Great Lakes, with migrations westward and into more northern latitudes, local adaptations within each lake basin may have led to selection at certain loci. Alternatively, large differences in allele frequency at a small subset of loci could be produced by genetic drift during range expansion from the lower Great Lakes to the upper Great Lakes (Excoffier, Foll, & Petit, 2009). Nonetheless, OutFLANK has been shown to have a low false‐positive rate relative to other outlier detection methods under range expansion scenarios (Whitlock & Lotterhos, 2015). Further attention to these putatively adaptive loci, others identified in future studies, and those previously observed in closely related taxa (Hess, Campbell, Close, Docker, & Narum, 2013) may help to shed light on the genetic basis for local adaptation in invasive and native sea lamprey populations.

Ordination of individuals in multivariate space (Figure 3) also revealed that larval sea lampreys from disparate locations can be genetically differentiated. As expected, individuals were largely separated by lake of origin, with individuals from Lake Ontario (DC) being separated from all other locations along the first DAPC axis and Lake Michigan and St. Clair River being separated from Lake Superior origin individuals along DAPC axis 2. Data from the SCR revealed that larvae from different cohorts cluster with different groups of adults from different streams (age‐2 larvae with adults from the St. Mary's River and age‐3 larvae with adults from the Carp River; Figure 3). These preliminary results are based on only two tributaries but suggest an intriguing explanation that the genetic similarities of larvae to adults collected from different streams can indicate the stream of origin of adults of each cohort. Collections of additional data would be important to better characterize lake‐level and stream‐level (year class) variation in allele frequencies. If such an explanation were supported, a large‐scale genetic sibship or parent‐offspring‐based tagging studies could allow managers to identify sea lamprey natal stream origins, which is the highest priority for the Great Lakes Sea Lamprey Control Program (SLCB Research Priorities, 2016). The feasibility and efficacy of genetic tagging studies to provide valuable management insights have been demonstrated across a broad range of species (Anderson & Garza, 2006; Bravington, Grewe, & Davies, 2016; De Barba et al., 2010). Importantly, genetic tagging studies can confidently identify full‐ and half‐sibling relationships without genotyping parents (Jones & Wang, 2010), especially when several hundred loci are genotyped (Santure et al., 2010) as demonstrated by simulated and empirical data shown here.

### 4.3 Analyses of simulated and empirical pedigrees

Simulations demonstrated that with 200 or 500 SNP loci separated by 2 MB across the genome, pedigrees could be established with high confidence, even with 1,000 adults contributing to simulated offspring (Figures 5 and 6). Given the size of the St. Clair River relative to Duffins Creek, it is not surprising that no related individuals were observed in the small samples from the St. Clair River. Furthermore, given the size of Duffins Creek, it is unlikely that the total number of successfully breeding adults is near 1,000. Thus, simulations suggest there are few pedigree misallocations present among the collection of inferred full and half‐siblings in the Duffins Creek pedigree.

The program LDNe estimates effective size based on a signature of linkage disequilibrium among loci. Thus, loci that are physically linked on the same chromosome may bias estimates of effective size when using LDNe (Waples et al., 2016). We sought to determine how much estimates of *N*_b_ differed when using all loci pairs in the RAPTURE panel or only loci pairs that were not physically linked. Importantly, results did not demonstrably change between the two approaches.The lack of change is likely due to the fact that lamprey have a large number of chromosomes and that for this set of loci, the number of intrachromosomal locus pairs is negligible relative to the number of interchromosomal pairs (Waples et al., 2016). Expansion of the number of loci for pedigree analyses would greatly increase statistical power to resolve pedigrees.

Given results in this study, a full suite of RAD loci may be utilized for pedigree reconstruction, further expanding the spatial and demographic scale that these loci may be used. We note that simulations were conducted assuming independent loci and with mean and variance in reproductive success lower than empirically estimated for the DC larval samples in this study (Table 4). Incidence of multiple SNPs per locus means that SNPs could be phased into microhaplotypes, which would potentially increase the number of alleles per locus and our ability to correctly resolve pedigrees (Baetscher et al., 2017). Results collectively suggest further simulation studies would be useful to better understand how violating assumptions regarding independent loci affect estimates of *N*_b_ and genetic pedigree reconstruction.

The main ecological findings in this study highlight several potential downstream application of this RAPTURE panel. First, we found significant variation in larval family distributions collected in DC across three years. Year‐to‐year consistency in areas of presumed high spawning activity based on colocation of many related individuals could be used to identify areas for established methods of adult suppression including trapping, use of pheromone attractants, and repellents. Additionally, dispersion during the larval stage is difficult to observe with mark–recapture due to requirements of (a) recapturing marked individuals after release and (b) assumption that capture, marking, releasing does not affect postrelease dispersal. The panel of loci developed as part of this study will enable greater efficiency to capture related individuals and thus study dispersal through the life cycle of sea lamprey. Results from our study show that larval lamprey families dispersed downstream across multiple cohorts. Given this information, it may be necessary to treat larger stream areas. Finally, our ability to detect large larval families in DC relative to our inability to detect any families in STC highlights the need to better understanding the sampling efforts necessary for streams of varying sizes and sea lamprey spawning populations. Such information will inform potential large‐scale close‐kin mark–recapture studies.

### 4.4 Effects of confounding factors

#### 4.4.1 Effects of potential aging error on pedigree inference and *N*_b_

Larval lamprey were aged based on established length–frequency distributions (Sethi et al., 2017). Aging error, particularly in tributaries of low productivity, can lead to individuals from multiple year cohorts being assigned to a single‐year class (Dawson et al., 2009). Because of the semelparous breeding with different adults producing each cohort, the most prudent approach would be tiered to (a) estimate the number of adults that produced the larvae present in a given year and then (b) separate adult year classes based on assumptions of size‐at‐age distributions, mortality rates, transformation rates, etc. Grouping offspring from different year classes will increase the numbers of adults contributing to offspring and would provide an adult abundance estimate representing multiple spawning years. Although useful for understanding long‐term trends in abundance per tributary, as well as better understanding the variability of stream use by spawning adults among years evaluated, such multiyear estimates of spawner abundance may not be useful to the sea lamprey control program because the program is managed based on annual assessment data. Effects of amalgamation of offspring from multiple year classes into a single‐year class may also affect estimates of mean and variance in adult reproductive success that will affect inferred adult number and effective number (*N*_b_) estimated using either LD (Waples et al., 2016) or family size (Wang, 2009). The problem of overlapping size distributions is not likely to affect age‐1 individuals because of greater size discrimination relative to size differences among members of older age classes.

#### 4.4.2 Effects of ascertainment bias

SNP genotyping panels are influenced by ascertainment, and the resulting biases might affect subsequent analyses (Lachance & Tishkoff, 2013). Biased ascertainment has been observed in SNP genotyping panels developed for model (Clark, Hubisz, Bustamante, Williamson, & Nielsen, 2005) and nonmodel species (Seeb, Templin, et al., 2011), specifically when ascertainment is conducted using few populations or individuals that are not representative of the genetic diversity encountered across the range (Seeb, Carvalho, et al., 2011). Our ascertainment process may have resulted in the detection of an excess of loci with intermediate minor allele frequencies in DC and the SCR. If present, our panel should include multiple low minor allele frequency variants that are private to DC and potentially nearby populations. The inclusion of multiple large full‐sibling families from DC in the discovery panel likely resulted in the disproportionate inclusion of low minor allele frequency loci that were segregating within the parents that produced the largest families. An excess of intermediate frequency alleles in the larger metapopulation and an excess of loci with alleles that are private to a few lineages within the DC population are expected to result in downwardly biased estimates of interpopulation variance and overestimation of variance within DC (Morin, Luikart, & Wayne, 2004). However, sea lamprey do not exhibit natal homing and thus adults spawning in a given stream are likely widely representative of individuals produced in streams across each lake basin. Further, population measures of diversity and average minor allele frequency based on sampling of adults from a geographically more expansive set of stream suggest that bias was not great.

The ascertainment process can have effects on conclusions drawn from principle components analysis (Albrechtsen, Nielsen, & Nielsen, 2010), while Bayesian clustering programs such as STRUCTURE have been shown to be relatively resilient to ascertainment bias (Haasl & Payseur, 2011). Interestingly, our DAPC depicts patterns of population structure similar to those described in previous studies conducted using microsatellites (Bryan et al., 2005). Assignment tests (Bradbury et al., 2011), parentage analysis, and individual identification are expected to be minimally affected by ascertainment bias because using markers with higher heterozygosity markers increases statistical power (Morin et al., 2004). Temporal F_ST_ and linkage disequilibrium‐derived estimates of effective population size are also expected to be minimally affected by ascertainment (Morin et al., 2004). Demographic inferences can likely be made after correcting for biases induced by ascertainment scheme (Wakeley, Nielsen, Liu‐Cordero, & Ardlie, 2001). Ascertainment bias has the potential to increase false‐positive rates for genome‐wide scans for selection (Lachance & Tishkoff, 2013).

### 4.5 Future applications for the RAD capture sea lamprey SNP array

With the large number of loci now available for sea lamprey, future applications in many areas of sea lamprey research and assessment are not limited by statistical power of the markers available, but by the ability to collect adequate sample sizes in the context of appropriate spatial scales. Data presented here represent one of the few studies to employ the RAD capture genotyping method for fish (others include Margres et al., 2018; Ali et al., 2016; Komoroske et al., 2019; review in Meek & Larson, 2019). Research outcomes from this project could provide technical assistance to sea lamprey researchers and managers involved in the control of invasive populations and the conservation of native populations.

Results from this project highlight several biological attributes of sea lamprey that can be used for future control and conservation efforts. The following represent a nonexhaustive set of examples of additional research and management questions that can be addressed with data from this RAD capture SNP panel.

1. Analyses of pedigrees from multiple year classes in DC demonstrate that estimates of the number of spawning adults and effective numbers of breeding adults that produced each cohort in a larval sample can be obtained. A useful next step would be to estimate the total river spawner abundance for each year (cohort‐specific), which would require additional analyses. One benefit to sea lamprey control of using pedigree analysis of larvae rather than mark–recapture of adults to estimate stream‐specific annual adult abundance is that any captured adults could be immediately removed from the system rather than marked and released (i.e., allowed to reproduce). Pedigree analysis of larvae could also provide estimates of adult abundance in rivers where traps are not operated (e.g., rivers lacking dams). One drawback to larval‐based adult abundance estimates, however, is that it requires a time lag (e.g., provides estimates one or more years after spawning occurred), whereas mark–recapture of adults provides information to managers during the year of spawning.
2. Identification of sibling sea lamprey across life stages (i.e., linking premetamorph juveniles in a stream to siblings sampled as spawning adults years later) could address one of the highest priorities of the sea lamprey control program: the ability to identify the stream of origin of the parasitic and adult lampreys in each lake. One benefit of genetic mark–recapture for stream origin questions is that marked individuals do not need to be handled; only their siblings. Thus, a sample of larvae collected during treatments or other outmigrant/larvae collections could be used to recapture (as adults or parasitic juveniles) their siblings. This seems to have at least the same power as coded wire tagging studies but does not rely on assumptions of handling effects and postrelease mortality (Johnson et al., 2014).
3. The sea lamprey assessment program could benefit from having the ability to characterize dispersion of larvae from the same full‐ and half‐sibling families. Previously, we have shown (Derosier et al., 2007; Gilmore, 2004) that larval sea lampreys disperse from nests downstream in a time‐dependent manner. Using multiple long‐term assessment streams, Gilmore (2004) demonstrated that full‐ and half‐sibling sea lampreys can colonize up to 5 km of stream from putative upstream nest locations by age 1. Data from DC in this study revealed dispersion of larvae from full‐sib families in sequential downstream sampling locales, providing evidence of where spawning was initiated and where downstream dispersal began. Sampling of cohorts in multiple years can provide measures of relative survival among age classes and habitats. For the lamprey emigrating from the stream as parasites, one hypothesis to examine would be whether outmigrating individuals represented a random sample of larvae collected during electrofishing or an over‐representation of sibling groups.
4. Pedigree data collected before and after lampricide treatment could provide estimates of lampricide efficiency in different stream areas, thereby identifying locations/habitats where treatments are more or less effective than others. Does lampricide kill all larvae equally across the full pedigree for a given stream or are some sibling groups disproportionally affected by lampricide treatment? Is there is a difference can it be explained by location of larvae in the stream when sampled—main channel versus backwater, still water versus riffle versus ground‐seep, etc.? By comparing individual genotypes sampled before and after pesticide treatment, one could find some families are over‐ or under‐represented, leading to at least two explanations: (a) Families occupy different habitats, and treatment effectiveness varies among habitats; and (b) families have different physiological tolerances to lampricides (i.e., dead in treatment vs. alive as residuals).
5. The RAPTURE panel described here may be used for similar efforts in the native range for sea lamprey. Large‐scale genetic parentage studies have provided several valuable insights into sea lamprey conservation efforts in native marine systems including estimating population abundance, evaluating environmental correlates of fitness, as well as estimating effective population size (e.g., Duong et al., 2013; Sard et al., 2016). Additionally, adaptive loci may be identified and used to inform which fish are reintroduced in specific locations to improve reintroduction outcomes.

## 5 CONFLICT OF INTERST

None declared.

## AUTHOR CONTRIBUTIONS

GB, JA. CH, PH, KT, and KS designed the research; GB, CH, PH, and KT were involved in field collection of samples; NS, SS, JH, and JK were responsible for laboratory analyses; and NS, SS, JH, and JA conducted bioinformatic and statistical analysis of molecular data and field data. All authors contributed to writing of the manuscript.

## Data Availability

All data and computational scripts necessary to perform the described analyses are available at http://www.github.com/ScribnerLab/SeaLampreyRapture. R scripts, custom functions, and R readable datafiles are available by downloading this project's research compendium presented as the SeaLampreyRAPTURE R package from the above GitHub repository. The GitHub repository has been archived using Zenodo and assigned a DOI (https://doi.org/10.5281/zenodo.3459520). Raw data files are available on the NCBI Short Read Archive [https://www.ncbi.nlm.nih.gov/sra/PRJNA579594]. Any use of trade, firm, or product names is for descriptive purposes only and does not imply endorsement by the US Government.

## References

[ece36001-bib-0001] Abadia‐Cardoso, A. , Anderson, E. C. , Pearse, D. E. , & Garza, J. C. (2013). Large‐scale parentage analysis reveals reproductive patterns and heritability of spawning timing in a hatchery population of steelhead (*Oncorhynchys mykiss*). Molecular Ecology, 22, 4733–4746. 10.1111/mec.12426 23962061

[ece36001-bib-0002] Albrechtsen, A. , Nielsen, F. C. , & Nielsen, R. (2010). Ascertainment biases in SNP chips affect measures of population divergence. Molecular Biology and Evolution, 27, 2534–2547. 10.1093/molbev/msq148 20558595 PMC3107607

[ece36001-bib-0003] Ali, O. A. , O'Rourke, S. M. , Amish, S. J. , Meek, M. H. , Luikart, G. , Jeffres, C. , & Miller, M. R. (2016). RAD Capture (Rapture): Flexible and efficient sequence‐based genotyping. Genetics, 202, 389–400. 10.1534/genetics.115.183665 26715661 PMC4788223

[ece36001-bib-0004] Allendorf, F. W. , Hohenlohe, P. A. , & Luikart, G. (2010). Genomics and the future of conservation genetics. Nature Reviews Genetics, 11, 697–709. 10.1038/nrg2844 20847747

[ece36001-bib-0005] Anderson, E. C. , & Garza, J. C. (2006). The power of single‐nucleotide polymorphisms for large‐scale parentage inference. Genetics, 172, 2567–2582. 10.1534/genetics.105.048074 16387880 PMC1456362

[ece36001-bib-0006] Andrews, S. (2014). FastQC a quality control tool for high throughput sequence data. Retrieved from http://www.bioinformatics.babraham.ac.uk/projects/fastqc/

[ece36001-bib-0007] Applegate, V. C. (1950). Natural history of the sea lamprey, *Petromyzon marinus* in Michigan. Special Science Report US Fish Wildlife Service, 55, 1–237.

[ece36001-bib-0008] Applegate, V. C. , Howell, J. H. , Moffett, J. W. , & Smith, M. A. (1961). Use of 3‐trifluormethyl‐4‐nitrophenol as a selective sea lamprey larvicide. Great Lakes Fishery Commission Technical Report, 1, 1–35.

[ece36001-bib-0009] Araki, H. , Waples, R. S. , Ardren, W. R. , Cooper, B. , & Blouin, M. S. (2007). Effective population size of steelhead trout: influence of variance in reproductive success, hatchery programs, and genetic compensation between life history forms. Molecular Ecology, 16, 953–966. 10.1111/j.1365-294X.2006.03206.x 17305853

[ece36001-bib-0010] Baetscher, D. S. , Anderson, E. C. , Gilbert‐Horvath, E. A. , Malone, D. P. , Saarman, E. T. , Carr, M. H. , & Garza, J. C. (2019). Dispersal of a nearshore marine fish connects marine reserves and adjacent fished areas along an open coast. Molecular Ecology, 28, 1611–1623. 10.1111/mec.15044 30739378

[ece36001-bib-0011] Baetscher, D. S. , Clemento, A. J. , Ng, T. C. , Anderson, E. C. , & Garza, J. C. (2017). Microhaplotypes provide increased power from short‐read DNA sequences for relationship inference. Molecular Ecology Resources, 22, 4733–4750. 10.1111/1755-0998.12737 29143457

[ece36001-bib-0012] Barth, J. M. I. , Berg, P. R. , Jonsson, P. R. , Bonanomi, S. , Corell, H. , Hemmer‐Hansen, J. , … André, C. (2017). Genome architecture enables local adaptation of Atlantic cod despite high connectivity. Molecular Ecology, 26, 4452–4466. 10.1111/mec.14207 28626905

[ece36001-bib-0013] Bartron, M. L. , Sard, N. M. , & Scribner, K. T. (2018). Evaluation of effective number of breeders and coancestry among progeny produced using common hatchery mating strategies. Transactions of the American Fisheries Society, 147, 185–194. 10.1002/tafs.10013

[ece36001-bib-0014] Beacham, T. D. , Wallace, C. , MacConnachie, C. , Jonsen, K. , McIntosh, B. , Candy, J. R. , … Withler, R. E. (2017). Population and individual identification of coho salmon in British Columbia through parentage‐based tagging and genetic stock identification: An alternative to coded‐wire tags. Canadian Journal of Fisheries and Aquatic Sciences, 74, 1391–1410. 10.1139/cjfas-2016-0452

[ece36001-bib-0015] Beamish, F. W. H. (1980). Biology of the North American anadromous sea lamprey, *Petromyzon marinus* . Canadian Journal of Fisheries and Aquatic Sciences, 37, 1924–1943. 10.1139/f80-233

[ece36001-bib-0016] Benjamini, Y. , & Hochberg, Y. (1995). Controlling the false discovery rate: A practical and powerful approach to multiple testing. Journal of the Royal Statistical Society: Series B (Methodological), 57, 289–300. 10.1111/j.2517-6161.1995.tb02031.x

[ece36001-bib-0017] Bergstedt, R. A. , & Seelye, J. G. (1995). Evidence for lack of homing by sea lampreys. Transactions of the American Fisheries Society, 124, 235–239. 10.1577/1548-8659(1995)124<0235:EFLOHB>2.3.CO;2

[ece36001-bib-0018] Bjerselius, R. , Li, W. , Teeter, J. H. , Seelye, J. G. , Johnsen, P. B. , Maniak, P. J. , … Sorensen, P. W. (2000). Direct behavioral evidence that unique bile acids released by larval sea lamprey (*Petromyzon marinus*) function as a migratory pheromone. Canadian Journal of Fisheries and Aquatic Sciences, 57, 557–569. 10.1139/f99-290

[ece36001-bib-0019] Blouin, M. (2003). DNA‐based methods for pedigree reconstruction and kinship analysis in natural populations. Trends in Ecology and Evolution, 18, 503–511. 10.1016/S0169-5347(03)00225-8

[ece36001-bib-0020] Bolger, A. M. , Lohse, M. , & Usadel, B. (2014). Trimmomatic: A flexible trimmer for Illumina Sequence Data. Bioinformatics, 30, 2114–2120. 10.1093/bioinformatics/btu170 24695404 PMC4103590

[ece36001-bib-0021] Bradbury, I. R. , Hubert, S. , Higgins, B. , Bowman, S. , Paterson, I. G. , Snelgrove, P. V. , & Bentzen, P. (2011). Evaluating SNP ascertainment bias and its impact on population assignment in Atlantic cod, *Gadus morhua* . Molecular Ecology Resources, 11, 218–225. 10.1111/j.1755-0998.2010.02949.x 21429176

[ece36001-bib-0022] Bravington, M. V. , Eveson, J. P. , Grewe, P. M. , & Davies, C. R. (2017). SBT close‐kin mark‐recapture with parent‐offspring and half‐sibling pairs: Update on genotyping, kin‐finding and model development. *ESC*, 1708, 12.

[ece36001-bib-0023] Bravington, M. V. , Grewe, P. M. , & Davies, C. R. (2016). Absolute abundance of southern bluefin tuna estimated by close‐kin mark‐recapture. Nature Communications, 7, 13162 10.1038/ncomms13162 PMC5114523 27841264

[ece36001-bib-0024] Bryan, M. B. , Libants, S. V. , Warrillow, J. A. , Li, W. , & Scribner, K. T. (2003). Polymorphic microsatellite markers for the sea lamprey, *Petromyzon marinus* . Conservation Genetics, 4, 113–116. 10.1023/A:1021808909811

[ece36001-bib-0025] Bryan, M. B. , Zalinski, D. , Filcek, K. R. , Libants, S. , Li, W. , & Scribner, K. T. (2005). Patterns of invasions and colonization of the sea lamprey (*Petromyzon marinus*) in North America as revealed by microsatellite genotypes. Molecular Ecology, 14, 3757–3773. 10.1111/j.1365-294x.2005.02716.x 16202094

[ece36001-bib-0026] Buchinger, T. J. , Siefkes, M. J. , Zielinski, B. S. , Brant, C. O. , & Li, W. (2015). Chemical cues and pheromones in the sea lamprey (*Petromyzon marinus*). Frontiers in Zoology, 12, 1–11. 10.1186/s12983-015-0126-9 26609313 PMC4658815

[ece36001-bib-0027] Campbell, N. R. , Harmon, W. , & Narum, S. R. (2015). Genotyping‐in‐thousands by sequencing (GT‐seq): A cost effective SNP genotyping method based on custom amplicon sequencing. Molecular Ecology Resources, 15, 855–867. 10.1111/1755-0998.12357 25476721

[ece36001-bib-0028] Catchen, J. , Amores, A. , Hohenlohe, P. , Cresko, W. , & Postlethwait, J. (2011). Stacks: Building and genotyping loci de novo from short‐read sequences. G3: Genes, Genomes, Genetics, 1, 171–182. 10.1534/g3.111.000240 22384329 PMC3276136

[ece36001-bib-0029] Catchen, J. , Hohenlohe, P. A. , Bassham, S. , Amores, A. , & Cresko, W. A. (2013). Stacks: An analysis tool set for population genomics. Molecular Ecology, 22, 3124–3140. 10.1111/mec.12354 23701397 PMC3936987

[ece36001-bib-0030] Christie, W. J. (1974). Changes in the fish species composition of the Great Lakes. Journal Fisheries Research Board of Canada, 31, 827–854. 10.1139/f74-104

[ece36001-bib-0031] Christie, G. C. , Adams, J. V. , Steeves, T. B. , Slade, J. W. , Cuddy, D. W. , Fodale, M. F. , … Jones, M. L. (2003). Selecting Great Lakes streams for lampricide treatment based on larval sea lamprey surveys. Journal of Great Lakes Research, 29, 152–160.

[ece36001-bib-0032] Clark, A. G. , Hubisz, M. J. , Bustamante, C. D. , Williamson, S. H. , & Nielsen, R. (2005). Ascertainment bias in studies of human genome‐wide polymorphism. Genome Research, 15, 1496–1502. 10.1101/gr.4107905 16251459 PMC1310637

[ece36001-bib-0033] Cockerham, C. C. (1967). Group inbreeding and coancestry. Genetics, 56, 89–95. 6035597 10.1093/genetics/56.1.89 PMC1211496

[ece36001-bib-0034] Cosart, T. , Beja‐Pereira, A. , Chen, S. , Ng, S. B. , Shendure, J. , & Luikart, G. (2011). Exome‐wide DNA capture and next generation sequencing in domestic and wild species. BMC Genomics, 12, 347 10.1186/1471-2164-12-347 21729323 PMC3146453

[ece36001-bib-0035] Danecek, P. , Auton, A. , Abecasis, G. , Albers, C. A. , Banks, E. , DePristo, M. A. , … Durbin, R. (2011). 1000 genomes project analysis group: The variant call format and VCFtools. Bioinformatics, 27, 2156–2158. 10.1093/bioinformatics/btr330 21653522 PMC3137218

[ece36001-bib-0036] Dawson, H. A. , Jones, M. L. , Scribner, K. T. , & Gilmore, S. A. (2009). An assessment of age determination methods for Great Lakes larval sea lampreys (*Petromyzon marinus*). North American Journal of Fisheries Management, 29, 914–927. 10.1577/M08-139.1

[ece36001-bib-0037] De Barba, M. , Waits, L. P. , Garton, E. O. , Genovesi, P. , Randi, E. , Mustoni, A. , & Groff, C. (2010). The power of genetic monitoring for studying demography, ecology and genetics of a reintroduced brown bear population. Molecular Ecology, 19, 3938–3951. 10.1111/j.1365-294X.2010.04791.x 20735733

[ece36001-bib-0038] Derosier, A. L. , Jones, M. L. , & Scribner, K. T. (2007). Dispersal of sea lamprey larvae during early life: Relevance for recruitment dynamics. Environmental Biology of Fishes, 78, 271–284. 10.1007/s10641-006-9095-3

[ece36001-bib-0039] Do, C. , Waples, R. S. , Peel, D. , Macbeth, G. M. , Tillett, B. J. , & Ovenden, J. R. (2014). NeEstimator V2: Re‐implementation of software for the estimation of contemporary effective population size (*N* _e_) from genetic data. Molecular Ecology Resources, 14, 209–214. 10.1111/1755-0998.12157 23992227

[ece36001-bib-0040] Dunlop, E. S. , McLaughlin, R. , Adams, J. V. , Jones, M. , Birceanu, O. , Christie, M. R. , … Wilkie, M. P. (2018). Rapid evolution meets invasive species control: The potential for pesticide resistance in sea lamprey. Canadian Journal of Fisheries and Aquatic Sciences, 75, 152–168. 10.1139/cjfas-2017-0015

[ece36001-bib-0041] Duong, T. Y. , Scribner, K. T. , Forsythe, P. S. , Crossman, J. A. , & Baker, E. A. (2013). Inter‐annual variation in effective number of breeders and estimation of effective population size in long‐lived iteroparous lake sturgeon (*Acipenser fulvescens*). Molecular Ecology, 22, 1282–1294. 10.1111/mec.12167 23293919

[ece36001-bib-0042] Ellner, S. , & Sasaki, A. (1996). Patterns of genetic polymorphism maintained by fluctuating selection with overlapping generations. Theoretical Population Biology, 50, 31–65. 10.1006/tpbi.1996.0022 8776837

[ece36001-bib-0043] Excoffier, L. , Foll, M. , & Petit, R. J. (2009). Genetic consequences of range expansions. Annual Review of Ecology, Evolution, and Systematics, 40, 481–501. 10.1146/annurev.ecolsys.39.110707.173414

[ece36001-bib-0044] Filcek, K. B. , Gilmore, S. A. , Jones, M. L. , & Scribner, K. T. (2005). Discriminating lamprey species using multi‐locus microsatellite genotypes. North American Journal of Fisheries Management, 25, 502–509. 10.1577/M03-206.1

[ece36001-bib-0045] Garcia de Leaniz, C. , Fleming, I. A. , Einum, S. , Verspoor, E. , Jordon, W. C. , Consuegra, S. , … Quinn, T. P. (2007). A critical review of adaptive genetic variation in Atlantic Salmon: Implications for conservation. Biological Reviews, 82, 173–211. 10.1111/j.1469-185X.2006.00004.x 17437557

[ece36001-bib-0046] Garrison, E. , & Marth, G. (2012). Haplotype‐based variant detection from short‐read sequencing. arXiv preprint arXiv:1207.3907 [q‐bio.GN.].

[ece36001-bib-0047] Gilmore, S. (2004). Genetic assessment of adult mating success and accuracy of statolith aging in the sea lamprey *Petromyzon marinus*. M.S. thesis, Michigan State University, Michigan.

[ece36001-bib-0048] Gingera, T. D. , Steeves, T. B. , Boguski, D. A. , Whyard, S. , Li, W. , & Docker, M. F. (2016). Detection and identification of lampreys in Great Lakes streams using environmental DNA. Journal of Great Lakes Research, 42, 649–659. 10.1016/j.jglr.2016.02.017

[ece36001-bib-0049] Goudet, J. (2004). hierfstat, a package for R to compute and test hierarchical F‐statistics. Molecular Ecology Resources, 5, 184–186. 10.1111/j.1471-8286.2004.00828.x

[ece36001-bib-0050] Haasl, R. J. , & Payseur, B. A. (2011). Multi‐locus inference of population structure: A comparison between single nucleotide polymorphisms and microsatellites. Heredity, 106, 158–171. 10.1038/hdy.2010.21 20332809 PMC2892635

[ece36001-bib-0051] Hand, B. K. , Hether, R. , Kovach, P. , Muhlfeld, C. C. , Amish, S. J. , Boyer, M. C. , … Luikart, G. (2015). Genomics and introgression: Discovery and mapping of thousands of species‐diagnostic SNPs using RAD sequencing. Current Zoology, 61, 146–154. 10.1093/czoolo/61.1.146

[ece36001-bib-0052] Hand, B. K. , Muhlfeld, C. C. , Wade, A. A. , Kovach, R. P. , Whited, D. C. , Narum, S. R. , … Luikart, G. (2016). Climate variables explain neutral and adaptive variation within salmonid metapopulations: The importance of replication in landscape genetics. Molecular Ecology, 25, 689–705. 10.1111/mec.13517 26677031

[ece36001-bib-0053] Hansen, M. J. , Madenjian, C. P. , Slade, J. W. , Steeves, T. B. , Almeida, P. R. , & Quintella, B. R. (2016). Population ecology of the sea lamprey (*Petromyzon marinus*) as an invasive species in the Laurentian Great Lakes and an imperiled species in Europe. Reviews in Fishery Biology and Fisheries, 26, 509–535. 10.1007/s11160-016-9440-3

[ece36001-bib-0054] Hess, J. E. , Campbell, N. R. , Close, D. A. , Docker, M. F. , & Narum, S. R. (2013). Population genomics of Pacific lamprey: Adaptive variation in a highly dispersive species. Molecular Ecology, 22, 2898–2916. 10.1111/mec.12150 23205767

[ece36001-bib-0055] Hess, J. E. , Campbell, N. R. , Docker, M. F. , Baker, C. , Jackson, A. , Lampman, R. , … Narum, S. R. (2015). Use of genotyping by sequencing data to develop a high‐throughput and multifunctional SNP panel for conservation applications in Pacific lamprey. Molecular Ecology Resources, 15, 187–202. 10.1111/1755-0998.12283 24842551

[ece36001-bib-0056] Hess, J. E. , Caudill, C. C. , Keefer, M. L. , McIlraith, B. J. , Moser, M. L. , & Narum, S. R. (2014). Genes predict long distance migration and large body size in a migratory fish, Pacific lamprey. Evolutionary Applications, 7, 1192–1208. 10.1111/eva.12203 25558280 PMC4275091

[ece36001-bib-0057] Hoffberg, S. L. , Kieran, T. J. , Catchen, J. M. , Devault, A. , Faircloth, B. C. , Mauricio, R. , & Glenn, T. C. (2016). RAD cap: Sequence capture of dual‐digest RAD seq libraries with identifiable duplicates and reduced missing data. Molecular Ecology Resources, 16, 1264–1278. 10.1111/1755-0998.12566 27416967

[ece36001-bib-0058] Hohenlohe, P. A. , Day, M. D. , Amish, S. J. , Miller, M. R. , Hughes, N. , Boyer, M. C. , … Luikart, G. (2013). Genomic patterns of introgression in rainbow and westslope cutthroat trout illuminated by overlapping paired‐end RAD sequencing. Molecular Ecology, 22, 3002–3013. 10.1111/mec.12239 23432212 PMC3664261

[ece36001-bib-0059] Hume, J. B. , Rechnagel, H. , Bean, C. W. , Adams, C. E. , & Mable, B. K. (2018). RADseq and mate choice assays reveal unidirectional gene flow among three lamprey ecotypes despite weak assortative mating: Insights into the formation and stability of multiple ecotypes in sympatry. Molecular Ecology, 27, 4572–4590. 10.1111/mec.14881 30252984

[ece36001-bib-0060] Johnson, N. S. , Swink, W. D. , Brenden, T. O. , Slade, J. W. , Steeves, T. B. , Fodale, M. F. , & Jones, M. L. (2014). Survival and metamorphosis of low‐density populations of larval sea lampreys (*Petromyzon marinus*) in streams following lampricide treatment. Journal of Great Lakes Research, 40, 155–163. 10.1016/j.jglr.2013.12.005

[ece36001-bib-0061] Jombart, T. (2008). adegenet: A R package for the multivariate analysis of genetic markers. Bioinformatics, 24, 1403–1405. 10.1093/bioinformatics/btn129 18397895

[ece36001-bib-0062] Jombart, T. , & Ahmed, I. (2011). adegenet 1.3‐1: New tools for the analysis of genome‐wide SNP data. Bioinformatics, 27, 3070–3071. 10.1093/bioinformatics/btr521 21926124 PMC3198581

[ece36001-bib-0063] Jombart, T. , Devillard, S. , & Balloux, F. (2010). Discriminant analysis of principal components: A new method for the analysis of genetically structured populations. BMC Genetics, 11, 94 10.1186/1471-2156-11-94 20950446 PMC2973851

[ece36001-bib-0064] Jones, O. R. , & Wang, J. (2010). COLONY: A program for parentage and sibship inference from multilocus genotype data. Molecular Ecology Resources, 10, 551–555. 10.1111/j.1755-0998.2009.02787.x 21565056

[ece36001-bib-0065] Kardos, M. , Luikart, G. , & Allendorf, F. W. (2015). Measuring individual inbreeding in the age of genomics: Marker‐based measures are better than pedigrees. Heredity, 115, 63–72. 10.1038/hdy.2015.17 26059970 PMC4815495

[ece36001-bib-0066] Kardos, M. , Taylor, H. R. , Ellegren, H. , Luikart, G. , & Allendorf, F. (2016). Genomics advances the study of inbreeding depression in the wild. Evolutionary Applications, 9, 1205–1218. 10.1111/eva.12414 27877200 PMC5108213

[ece36001-bib-0067] Komoroske, L. , Miller, M. , O'Rourke, S. , Stewart, K. R. , Jensen, M. P. , & Dutton, P. H. (2019). A versatile rapture (RAD‐Capture) platform for genotyping marine turtles. Molecular Ecology Resources, 19, 497–511. 10.1111/1755-0998.12980 30576074

[ece36001-bib-0068] Krawczak, M. (1999). Informativity assessment for biallelic single nucleotide polymorphisms. Electrophoresis, 20, 1676–1681. 10.1002/(SICI)1522-2683(19990101)20:8<1676:AID-ELPS1676>3.0.CO;2-D 10435431

[ece36001-bib-0069] Krumm, N. , Turner, T. N. , Baker, C. G. , Vives, L. , Mohajeri, K. , Witherspoon, K. T. , … Eichler, E. E. (2015). Excess of rare, inherited truncating mutations in autism. Nature Genetics, 47, 582–588. 10.1038/ng.3303 25961944 PMC4449286

[ece36001-bib-0070] Lachance, J. , & Tishkoff, S. A. (2013). SNP ascertainment bias in population genetic analyses: Why it is important, and how to correct it. BioEssays, 35, 780–786. 10.1002/bies.201300014 23836388 PMC3849385

[ece36001-bib-0071] Lawrie, A. H. (1970). The sea lamprey in the Great Lakes. Transactions American Fisheries Society, 4, 766–774. 10.1577/1548-8659(1970)99<766:TSLITG>2.0.CO;2

[ece36001-bib-0072] Lee, W. J. , & Kocher, T. D. (1995). Complete sequence of a sea lamprey (*Petromyzon marinus*) mitochondrial genome: Early establishment of the vertebrate genome organization. Genetics, 139, 873–887. 7713438 10.1093/genetics/139.2.873 PMC1206387

[ece36001-bib-0073] Li, H. (2013). Aligning sequence reads, clone sequences and assembly contigs with BWA‐MEM. arXiv:1303.3997v1 [q‐bio.GN]. DOI: 10.6084/m9.figshare.963153.v1.

[ece36001-bib-0074] Li, H. , Handsaker, B. , Wysoker, A. , Fennell, T. , Ruan, J. , Homer, N. … 1000 Genome Project Data Processing Subgroup (2009). The Sequence alignment/map (SAM) format and SAMtools. Bioinformatics, 25, 2078–2079. 10.1093/bioinformatics/btp352 19505943 PMC2723002

[ece36001-bib-0075] Luikart, G. , England, P. R. , Tallmon, D. , Jordon, S. , & Taberlet, P. (2003). The power and promise of population genomics: From genotyping to genome typing. Nature Reviews Genetics, 4, 981–987. 10.1038/nrg1226 14631358

[ece36001-bib-0076] Manion, P. J. , & Hanson, D. A. (1980). Spawning behavior and fecundity of lampreys from the upper three Great Lakes. Canadian Journal of Fisheries and Aquatic Sciences, 37, 1635–1640. 10.1139/f80-211

[ece36001-bib-0077] Margres, M. J. , Jones, M. E. , Epstein, B. , Kerlin, D. H. , Comte, S. , Fox, S. , & Lazenby, B. (2018). Large‐effect loci affect survival in Tasmanian devils (*Sarcophilus harrisii*) infected with a transmissible cancer. Molecular Ecology, 27, 4189–4199. 10.1111/mec.14853 30171778 PMC6759049

[ece36001-bib-0078] Maruki, T. , & Lynch, M. (2017). Genotype calling from population‐genomic sequencing data. G3: Genes, Genomes, and Genetics, 7, 1393–1404. 10.1534/g3.117.039008 PMC5427492 28108551

[ece36001-bib-0079] Mateus, C. S. , Rodríguez‐Muñoz, R. , Quintella, B. R. , Alves, M. J. , & Almeida, P. R. (2012). Lampreys of the Iberian Peninsula: Distribution, population status and conservation. Endangered Species Research, 16, 183–198. 10.3354/esr00405

[ece36001-bib-0080] McCauley, D. W. , Docker, M. F. , Whyard, S. , & Li, W. (2015). Lampreys as diverse model organisms in the genomics era. BioScience, 65, 1046–1057. 10.1093/biosci/biv139 26951616 PMC4777059

[ece36001-bib-0081] McKinney, G. J. , Waples, R. K. , Seeb, L. W. , & Seeb, J. E. (2017). Paralogs are revealed by proportion of heterozygotes and deviations in read ratios in genotyping‐by‐sequencing data from natural populations. Molecular Ecology Resources, 17, 656–669. 10.1111/1755-0998.12613 27762098

[ece36001-bib-0082] Meek, M. H. , & Larson, W. A. (2019). The future is now: Amplicon sequencing and sequence capture usher in the conservation genomics era. Molecular Ecology Resources, 19(4), 795–803. 10.1111/1755-0998.12998 30681776

[ece36001-bib-0083] Morin, P. A. , Luikart, G. , & Wayne, R. K. (2004). SNPs in ecology, evolution and conservation. Trends in Ecology and Evolution, 19, 208–216. 10.1016/j.tree.2004.01.009

[ece36001-bib-0084] Morman, R. H. , Cuddy, D. W. , & Rugen, P. C. (1980). Factors influencing the distribution of sea lamprey (*Petromyzon marinus*) ammocoetes and metamorphosed individuals. Canadian Journal of Fisheries and Aquatic Sciences, 37, 1811–1826. 10.1139/f80-211

[ece36001-bib-0085] Mullett, K. M. , Heinrich, J. W. , Adams, J. V. , Young, R. J. , Henson, M. P. , McDonald, R. B. , & Fodale, M. F. (2003). Estimating lake‐wide abundance of spawning‐phase sea lampreys (*Petromyzon marinus*) in the Great Lakes: Extrapolating from sampled streams usingregression models. Journal of Great Lakes Research, 29, 240–252.

[ece36001-bib-0086] Nei, M. (1973). Analysis of gene diversity in subdivided populations. Proceedings National Academy of Sciences of the United States of America, 70, 3321–3323. 10.1073/pnas.70.12.3321 PMC427228 4519626

[ece36001-bib-0087] Oleksyk, T. K. , Smith, M. W. , & O'Brien, S. J. (2010). Genome‐wide scans for footprints of natural selection. Philosophical Transactions of the Royal Society B: Biological Sciences, 365(1537), 185–205. 10.1098/rstb.2009.0219 PMC2842710 20008396

[ece36001-bib-0088] Oosting, J. , Eilers, P. , & Menezes, R. (2018). quantsmooth: Quantile smoothing and genomic visualization of array data. R package version 1.48.0. 10.18129/B9.bioc.quantsmooth

[ece36001-bib-0089] Paris, J. , Stevens, J. R. , & Catchen, J. M. (2017). Lost in parameter space: A road map for stacks. Methods in Ecology and Evolution, 8, 1360–1373. 10.1111/2041-210X.12775

[ece36001-bib-0090] Prince, D. J. , O'Rourke, S. M. , Thompson, T. Q. , Ali, O. A. , Lyman, H. S. , Saglam, I. K. , … Miller, M. R. (2017). The evolutionary basis of premature migration in Pacific salmon highlights the utility of genomics for informing conservation. Science Advances, 3, e1603198 10.1126/sciadv.1603198 28835916 PMC5559211

[ece36001-bib-0091] Quinlan, A. R. , & Hal, I. M. (2010). BEDTools: A flexible suite of utilities for comparing genomic features. Bioinformatics, 26, 841–842. 10.1093/bioinformatics/btq033 20110278 PMC2832824

[ece36001-bib-0092] R Core Team (2018). R: A language and environment for statistical computing. Vienna, Austria: R Foundation for Statistical Computing.

[ece36001-bib-0093] Rodrıguez‐Munoz, R. , & Tregenza, T. (2009). Genetic compatibility and hatching success in the sea lamprey (*Petromyzon marinus*). Biological Letters, 5, 286–288. 10.1098/rsbl.2008.0650 PMC2665819 19049954

[ece36001-bib-0094] Rougemont, Q. , Gaigher, A. , Lasne, E. , Côte, J. , Coke, M. , Besnard, A.‐L. , … Evanno, G. (2015). Low reproductive isolation and highly variable levels of gene flow reveal limited progress towards speciation between European river and brook lampreys. Journal of Evolutionary Biology, 28, 2248–2263. 10.1111/jeb.12750 26348652

[ece36001-bib-0095] Rutter, M. A. , & Bence, J. R. (2003). An improved method to estimate sea lamprey wounding rate on hosts with application to lake trout in Lake Huron. Journal of Great Lakes Research, 29, 320–331. 10.1016/S0380-1330(03)70497-3

[ece36001-bib-0096] Santure, A. W. , Stapley, J. , Ball, A. D. , Birkhead, T. R. , Burke, T. , & Slate, J. (2010). On the use of large marker panels to estimate inbreeding and relatedness: Empirical and simulation studies of a pedigreed zebra finch population typed at 771 SNPs. Molecular Ecology, 19, 1439–1451. 10.1111/j.1365-294X.2010.04554.x 20149098

[ece36001-bib-0097] Sard, N. M. , Johnson, M. A. , Jacobson, D. P. , Hogansen, M. J. , O'Malley, K. G. , & Banks, M. A. (2016). Genetic monitoring guides adaptive management of a migratory fish reintroduction program. Animal Conservation, 19, 570–577. 10.1111/acv.12278

[ece36001-bib-0098] Savolainen, O. , Lascoux, M. , & Merila, J. (2013). Ecological genomics of local adaptation. Nature Reviews Genetics, 14, 807–820. 10.1038/nrg3522 24136507

[ece36001-bib-0099] Sea Lamprey Control Board (2016). Research priorities. Great Lakes Fishery Commission Retrieved from http://www.glfc.org/pubs/pdfs/research/2016_SLCB_research_priorities_FINAL.pdf

[ece36001-bib-0100] Seeb, J. E. , Carvalho, G. , Hauser, L. , Naish, K. , Roberts, S. , & Seeb, L. W. (2011). Single‐nucleotide polymorphism (SNP) discovery and applications of SNP genotyping in nonmodel organisms. Molecular Ecology Resources, 11, 1–8. 10.1111/j.1755-0998.2010.02979.x 21429158

[ece36001-bib-0101] Seeb, L. W. , Templin, W. D. , Sato, S. , Abe, S. , Warheit, K. , Park, J. Y. , & Seeb, J. E. (2011). Single nucleotide polymorphisms across a species' range: Implications for conservation studies of Pacific salmon. Molecular Ecology Resources, 11, 195–217. 10.1111/j.1755-0998.2010.02966.x 21429175

[ece36001-bib-0102] Sethi, S. A. , Gerken, J. , & Ashline, J. (2017). Accurate aging of juvenile salmonids using fork lengths. Fisheries Research, 185, 161–168. 10.1016/j.fishres.2016.09.012

[ece36001-bib-0103] Smith, B. R. , & Tibbles, J. J. (1980). Sea lamprey (*Petromyzon marinus*) in Lakes Huron, Michigan, and Superior: History of invasion and control, 1936–78. Canadian Journal of Fisheries and Aquatic Sciences, 37, 1780–1801. 10.1139/f80-222

[ece36001-bib-0104] Smith, J. J. , Timoshevskaya, N. , Ye, C. , Holt, C. , Keinath, M. C. , Parker, H. J. , … Amemiya, C. T. (2018). The sea lamprey germline genome provides insights into programmed genome rearrangement and vertebrate evolution. Nature Genetics, 50, 270 10.1038/s41588-017-0036-1 29358652 PMC5805609

[ece36001-bib-0105] Sorensen, P. W. , & Vrieze, L. A. (2003). The chemical ecology and potential application of the sea lamprey migratory pheromone. Journal of Great Lakes Research, 29, 66–84. 10.1016/S0380-1330(03)70478-X

[ece36001-bib-0106] Steele, C. A. , Anderson, E. C. , Ackerman, M. W. , Hess, M. A. , Campbell, N. R. , Narum, S. R. , & Cambell, M. R. (2013). A validation of parentage‐based tagging using hatchery steelhead in the Snake River basis. Canadian Journal of Fisheries and Aquatic Sciences, 70, 1046–1054. 10.1139/cjfas-2012-0451

[ece36001-bib-0107] Strucken, E. M. , Lee, S. H. , Lee, H. K. , Song, K. D. , Gibson, J. P. , & Gondro, C. (2016). How many markers are enough? Factors influencing parentage testing in different livestock populations. Journal of Animal Breeding and Genetics, 133, 13–23. 10.1111/jbg.12179 26234440

[ece36001-bib-0108] Sullivan, P. , Adair, R. , & Woldt, A. (2016). Sea lamprey control in the Great Lakes 2015. Annual Report to the Great Lakes Fishery Commission, Ann Arbor, MI Retrieved from http://www.glfc.org/pubs/slcp/annual_reports/ANNUAL_REPORT_2015.pdf

[ece36001-bib-0109] Wakeley, J. , Nielsen, R. , Liu‐Cordero, S. N. , & Ardlie, K. (2001). The discovery of single‐nucleotide polymorphisms—and inferences about human demographic history. The American Journal of Human Genetics, 69, 1332–1347. 10.1086/324521 11704929 PMC1235544

[ece36001-bib-0110] Waldman, J. R. , Grunwald, C. , Roy, N. K. , & Wirgin, I. I. (2004). Mitochondrial DNA analysis indicates sea lam‐preys are indigenous to Lake Ontario. Transactions of the American Fisheries Society, 133, 950–960. 10.1577/T08-035R.1

[ece36001-bib-0111] Wang, J. (2004). Sibship reconstruction from genetic data with typing errors. Genetics, 166, 1963–1979. 10.1534/genetics.166.4.1963 15126412 PMC1470831

[ece36001-bib-0112] Wang, J. (2009). A new method for estimating effective population sizes from a single sample of multilocus genotypes. Molecular Ecology, 18, 2148–2164. 10.1111/j.1365-294X.2009.04175.x 19389175

[ece36001-bib-0113] Wang, J. (2013). A simulation module in the computer program COLONY for sibship and parentage analysis. Molecular Ecology Resources, 13, 734–739. 10.1111/1755-0998 23615269

[ece36001-bib-0114] Waples, R. S. (2010). Spatial‐temporal stratifications in natural populations and how they affect understanding and estimation of effective population size. Molecular Ecology Resources, 10, 785–796. 10.1111/j.1755-0998.2010.02876.x 21565090

[ece36001-bib-0115] Waples, R. S. , & Do, C. (2008). LDNE: A program for estimating effective population size from data on linkage disequilibrium. Molecular Ecology Resources, 8, 753–756. 10.1111/j.1755-0998.2007.02061.x 21585883

[ece36001-bib-0116] Waples, R. K. , Larson, W. A. , & Waples, R. S. (2016). Estimating contemporary effective population size in non‐model species using linkage disequilibrium across thousands of loci. Heredity, 117, 233–240. 10.1038/hdy.2016.60 27553452 PMC5026758

[ece36001-bib-0117] Waples, R. S. , Scribner, K. T. , Moore, J. , Draheim, H. , Etter, D. , & Boersen, M. (2018). Accounting for age structure and spatial structure in eco‐evolutionary analyses of a large, mobile vertebrate. Journal of Heredity, 109, 709–723. 10.1093/jhered/esy018 29668993

[ece36001-bib-0118] Whitlock, M. C. , & Lotterhos, K. E. (2015). Reliable detection of loci responsible for local adaptation: Inference of a null model through trimming the distribution of FST. American Naturalist, 186, S24–S36. 10.1086/682949 26656214

[ece36001-bib-0119] Wickham, H. (2016). ggplot2: Elegant graphics for data analysis. New York, NY: Springer.

[ece36001-bib-0120] Wickham, H. (2017). tidyverse: Easily Install and Load the 'Tidyverse'. R package version 1. Retrieved from https://CRAN.R-project.org/package=tidyverse

[ece36001-bib-0121] Winter, D. J. (2012). MMOD: an R library for the calculation of population differentiation statistics. Molecular ecology resources, 12(6), 1158–1160. 22883857 10.1111/j.1755-0998.2012.03174.x

